# A Molecular Modeling Approach to Identify Potential Antileishmanial Compounds Against the Cell Division Cycle (cdc)-2-Related Kinase 12 (CRK12) Receptor of *Leishmania donovani*

**DOI:** 10.3390/biom11030458

**Published:** 2021-03-18

**Authors:** Emmanuel Broni, Samuel K. Kwofie, Seth O. Asiedu, Whelton A. Miller, Michael D. Wilson

**Affiliations:** 1 Department of Biomedical Engineering, School of Engineering Sciences, College of Basic & Applied Sciences, University of Ghana, Legon, Accra LG 77, Ghana; ebroni002@st.ug.edu.gh; 2 West African Center for Cell Biology of Infectious Pathogens, Department of Biochemistry, Cell and Molecular Biology, College of Basic and Applied Sciences, University of Ghana, Legon, Accra LG 54, Ghana; 3 Department of Parasitology, Noguchi Memorial Institute for Medical Research (NMIMR), College of Health Sciences (CHS), University of Ghana, Legon, Accra LG 581, Ghana; sethasieduosei@gmail.com (S.O.A.); MWilson@noguchi.ug.edu.gh (M.D.W.); 4 Department of Medicine, Loyola University Medical Center, Maywood, IL 60153, USA; wmiller6@luc.edu; 5 Department of Molecular Pharmacology and Neuroscience, Loyola University Medical Center, Maywood, IL 60153, USA; 6 Department of Chemical and Biomolecular Engineering, School of Engineering and Applied Science, University of Pennsylvania, Philadelphia, IL 19104, USA

**Keywords:** Leishmaniasis, *Leishmania donovani*, CRK12, molecular docking, molecular dynamics simulation, natural products, leishmanicide

## Abstract

The huge burden of leishmaniasis caused by the trypanosomatid protozoan parasite *Leishmania* is well known. This illness was included in the list of neglected tropical diseases targeted for elimination by the World Health Organization. However, the increasing evidence of resistance to existing antimonial drugs has made the eradication of the disease difficult to achieve, thus warranting the search for new drug targets. We report here studies that used computational methods to identify inhibitors of receptors from natural products. The cell division cycle-2-related kinase 12 (CRK12) receptor is a plausible drug target against *Leishmania donovani*. This study modelled the 3D molecular structure of the *L. donovani* CRK12 (*Ld*CRK12) and screened for small molecules with potential inhibitory activity from African flora. An integrated library of 7722 African natural product-derived compounds and known inhibitors were screened against the *Ld*CRK12 using AutoDock Vina after performing energy minimization with GROMACS 2018. Four natural products, namely sesamin (NANPDB1649), methyl ellagic acid (NANPDB1406), stylopine (NANPDB2581), and sennecicannabine (NANPDB6446) were found to be potential *Ld*CRK12 inhibitory molecules. The molecular docking studies revealed two compounds NANPDB1406 and NANPDB2581 with binding affinities of −9.5 and −9.2 kcal/mol, respectively, against *Ld*CRK12 which were higher than those of the known inhibitors and drugs, including GSK3186899, amphotericin B, miltefosine, and paromomycin. All the four compounds were predicted to have inhibitory constant (Ki) values ranging from 0.108 to 0.587 μM. NANPDB2581, NANPDB1649 and NANPDB1406 were also predicted as antileishmanial with Pa and Pi values of 0.415 and 0.043, 0.391 and 0.052, and 0.351 and 0.071, respectively. Molecular dynamics simulations coupled with molecular mechanics Poisson–Boltzmann surface area (MM/PBSA) computations reinforced their good binding mechanisms. Most compounds were observed to bind in the ATP binding pocket of the kinase domain. Lys488 was predicted as a key residue critical for ligand binding in the ATP binding pocket of the *Ld*CRK12. The molecules were pharmacologically profiled as druglike with inconsequential toxicity. The identified molecules have scaffolds that could form the backbone for fragment-based drug design of novel leishmanicides but warrant further studies to evaluate their therapeutic potential.

## 1. Introduction

Leishmaniasis is a worldwide menace that exists in all continents except Oceania and it is endemic in the tropical and subtropical areas in Eastern Africa, Southern Europe, the Middle East, South-eastern Mexico, and Central and South America [1]. Approximately one million new cases and between 26,000 to 65,000 deaths occur annually [2]. Leishmaniasis is a neglected tropical disease caused by the trypanosomatid protozoan *Leishmania* parasites transmitted to humans through the bites of infected phlebotomine sand flies [3,4,5,6,7]. The disease manifests in three major forms, namely cutaneous leishmaniasis (CL), mucocutaneous leishmaniasis (MCL), and visceral leishmaniasis (VL) [8,9]. During the last decade, leishmaniasis has been observed with cases of co-infections in areas including the Mediterranean region, France, Italy, Portugal, Spain, Thailand, and Brazil [10,11,12]. Moreover, VL co-infection with HIV-infected patients living in Asia (especially India) and some African countries have been reported [13].

Leishmaniasis mostly affects people living in poor areas and places further economic stress on scanty financial resources [14,15,16]. The savings of most households are depleted to get treatment, while the few others incur debt. Leishmaniasis impacts negatively on the psychological and social status of infected persons. The disfiguring scars lead to various forms of social stigmatization and exclusion from community activities [17].

Currently, the dearth of effective and affordable drugs is a major problem hindering the eradication of leishmaniasis. Existing drugs are expensive, ranging from USD 30 to 1500 [17]. Paromomycin (PM) is the cheapest option in India, while liposomal amphotericin B (AmBisome) and miltefosine (Milt) costs USD 162–229 and USD 119 per patient, respectively [18].

Drug resistance is also a major issue facing the existing therapeutic options, hence the need to identify new drug targets. The cell division cycle (CDC)-2-related kinases CRK3, CRK6, and CRK12, which are cyclin-dependent kinases (CDKs) have recently been identified as plausible targets [19,20]. The overexpression of both CRK12 and the cyclin protein CYC9 have been reported to increase the resistance of *L. donovani* to pyrazolopyrimidines [20]. However, CRK12 has been reported to exist in a complex with CYC9 [19,20,21]. In bloodstream trypanosomes, both CRK12 and CYC9 are critical for proliferation in vitro [21]. Computational modelling studies showed that the most promising compound (GSK3186899), which inhibited the *L. donovani* parasites in a mouse model, binds to the CRK12 in the ATP binding pocket [19,20]. Mutation studies also suggested that GSK3186899 binds to CRK12 and not CYC9 since the effectiveness of GSK3186899 was reduced in a mutant version of the CRK12 [19,20]. The CRK12 is an essential gene for *L. donovani* and *Leishmania mexicana* promastigotes [20,22] and critical in the bloodstream stage of *Trypanosoma brucei* [21]. It also plays an essential role in the survival of trypanosomatids of *Trypanosoma brucei* [21], which corroborates CRK12 as a drug target for parasitic kinetoplastids belonging to the *Trypanosoma* genus [20,22]. In addition, the depletion of CRK12 results in the expansion of the flagellar pocket and impairment of endocytosis [21,23].

Computer-aided drug design (CADD) has become more advantageous than the traditional approach of high-throughput screening (HTS) as it has helped reduce the wastage of resources in terms of cost, effort, and time by significantly decreasing the number of compounds and filtering out only hits for further HTS. Natural products remain an untapped reservoir of new drug candidates for combating various kinds of diseases. The African flora is rich in biodiversity [24] and can be exploited to produce novel drug candidates from their natural sources. Therefore, the identification of new bioactive compounds via in silico drug design is vital in unravelling novel leads that have the potential to inhibit the activity of *L. donovani* by targeting the *Leishmania donovani* cell division cycle (CDC)-2-related kinase 12 (*Ld*CRK12).

This study seeks to model a reasonably accurate 3D structure of the *Ld*CRK12 and identify potential natural product-derived *Ld*CRK12 inhibitory compounds through virtual screening. It also sought to characterize the mechanisms of binding between the *Ld*CRK12 and potential inhibitory molecules using molecular dynamics (MDs) simulations integrated with molecular mechanics Poisson–Boltzmann surface area (MM/PBSA) [25,26]. In addition, it undertakes predictive pharmacokinetic and physicochemical profiling as well as the biological activity of compounds to identify potential novel drug-like leads.

## 2. Materials and Methods

**Figure 1:**
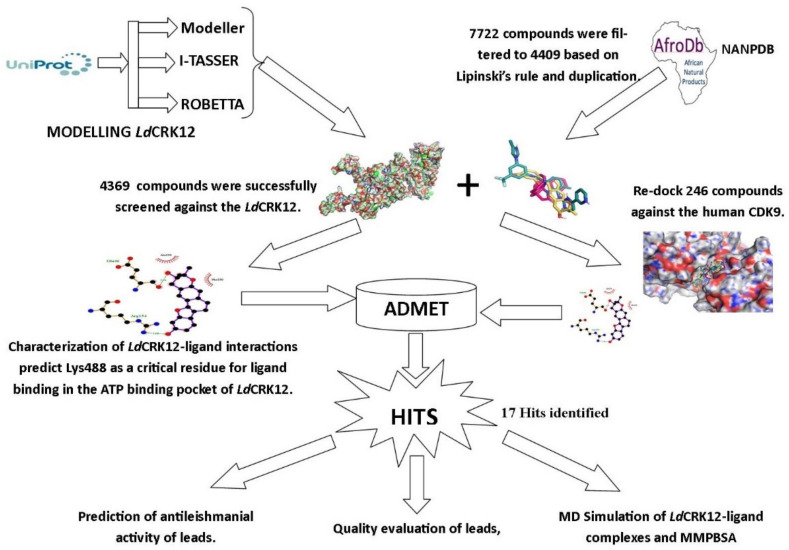
Methodology schema employed in this study for predicting potential antileishmanial compounds. Three modelling techniques comprising Modeller [29,30], I-TASSER [31,32,33,34] and Robetta [35,36,37] were used to predict potential *Ld*CRK12 structures. Evaluation of the predicted protein structures revealed the reasonably best model. Natural compounds from the African Natural Product Database (AfroDB), as well as the North African Natural Product Database (NANPDB) and known antileishmanial compounds, were docked against *Ld*CRK12 and the human CDK9 receptors. The potential lead compounds were subjected to absorption, distribution, metabolism, excretion, and toxicity (ADMET), biological activity predictions, and molecular dynamics (MDs) computations.

A schematic pipeline detailing the step-by-step techniques employed in the study is described in Figure 1. After modelling and validating the 3D structure of the *Ld*CRK12, structure-based virtual screening (SBVS) was performed to identify compounds with high binding affinity to the *Ld*CRK12 protein. Additionally, the selected hits were docked against the human cyclin-dependent kinase 9 (CDK9) since it is a homologue of the kinase domain of the *Ld*CRK12. Molecular interactions between the receptors and the compounds were investigated using MD studies including MM/PBSA. Chemical absorption, distribution, metabolism, excretion, and toxicity (ADMET) predictions were performed to evaluate the toxicity of the compounds. Thereafter, the biological activity of identified biomolecules was predicted using machine learning-based Open Bayesian techniques [27,28].

### 2.1. Sequence Retrieval

Since the experimental 3D structure of the *Ld*CRK12 does not exist, there was the need to employ modelling techniques to predict a reasonably accurate structure. The protein sequence of the *Ld*CRK12 with UniProtKB ID: A0A3S7WQK2 was retrieved from UniProtKB, a repository for amino acid sequences of proteins [38,39,40].

### 2.2. Obtaining the Structure of LdCRK12 and Human CDK9

Three different modelling approaches were employed in this study, comprising I-TASSER Suite [31,32,33,34], Robetta [35,36,37] and Modeller 9.20 [29,30] to predict the 3D structures of the *Ld*CRK12 protein. The structure of the human CDK9 was retrieved from the protein data bank (PDB) with PDB ID 4BCF. The details used to generate a reasonably valid structure of the *Ld*CRK12 via Modeller 9.20, I-TASSER, and Robetta are described.

#### 2.2.1. Template Search and Selection

The sequence of the *Ld*CRK12 was uploaded into SWISS-MODEL which performed a basic local alignment search tool (BLAST) search to obtain suitable templates that were identical to the target sequence [41]. A BLAST search was also conducted on the kinase domain using the BLAST option in UniProtKB. The most suitable template was then selected for modelling. 

#### 2.2.2. Structure Prediction Using Modeller

EasyModeller 4.0 [42], a graphical user interface (GUI) for Modeller was used to model the structure of *Ld*CRK12. The sequence and the selected template (PDB ID 4BCF) were imported into EasyModeller 4.0. Sequence alignment was performed to predict the secondary structure of the protein by using the selected template and the sequence of the *Ld*CRK12. Modeller then used the outcome to generate five models from which the best is selected based on their discrete optimized protein energy (DOPE) scores. DOPE is a statistical potential score used to evaluate homology models in protein structure prediction. For the same target, the model with the lowest DOPE score was chosen as the best [30,43].

#### 2.2.3. Structure Prediction Using I-TASSER

I-TASSER (https://zhanglab.ccmb.med.umich.edu/I-TASSER/; accessed on 22 October 2019) was employed to predict the structure of *Ld*CRK12. The amino acid sequence of the *Ld*CRK12 protein was uploaded into the I-TASSER platform and 5 protein structures were predicted using default parameters.

#### 2.2.4. Structure Prediction Using Robetta

The *Ld*CRK12 amino acid sequence was also uploaded into Robetta (https://robetta.bakerlab.org; accessed on 27 February 2020) and the “comparative modelling (CM) only” option was selected. Robetta then parsed the sequence into putative domains and built models for the domains which are homologues to solved protein structures using comparative modelling [37]. Five protein structures were predicted using default parameters.

### 2.3. Structural Validation

The quality of the generated models was assessed via SAVESv5.0 (http://servicesn.mbi.ucla.edu/SAVES/; accessed on 9 March 2020) along with Ramachandra plots from PROCHECK (https://www.ebi.ac.uk/thornton-srv/databases/pdbsum/Generate.html; accessed on 6 February 2021) [44]. The *z*-score obtained from ProSA-web [45,46], an indication of the overall model quality of the structures, was also determined. The z-score determines whether the input model is of X-ray or NMR quality. The local model quality of the structures was also determined by plotting the energies as a function of protein residue position. The positive values signify problematic or erroneous parts of the input structure. The reasonably best structure was selected based on the quality assessments performed.

### 2.4. Prediction of Binding Sites

Computed Atlas of Surface Topography of proteins (CASTp) [47,48] was used to predict potential binding sites of the *Ld*CRK12 protein. Chimera and PyMOL were used to assess the features of the predicted binding sites [49,50,51].

### 2.5. Preparation of Proteins and Ligand Libraries

The ligands were obtained from the African Natural Product Database (AfroDB) and the North African Natural Product Database (NANPDB) [52,53]. A total of 6842 compounds were obtained in 2D spatial data file (sdf) format from the NANPDB and were converted to 3D structures using Open Babel’s “gen3d” option. Additionally, 880 compounds were retrieved from the AfroDB in 3D sdf format. A total of 7722 compounds were obtained for this study by combining the two databases and removing duplicates. Additionally, the compound libraries were filtered based on Lipinski’s rule of five.

Compounds labelled 5, 7, and 8 which showed very good half-maximum effective concentration (EC_50_) values in a mouse model of visceral leishmaniasis by inhibiting CRK12 were used in this study [20]. Amphotericin B, miltefosine, and paromomycin were also included in the study. GSK3186899 (also known as compound 7 or DDD85365), amphotericin B, miltefosine, and paromomycin were retrieved from PubChem with compound identifiers (CIDs) 122429808, 5280965, 3599, and 165580, respectively. MarvinSketch 17.17.0 was used to generate the 3D sdf of compounds 5 and 8. Additionally, a 2-amino-4-heteroaryl-pyrimidine inhibitor (Code: T6Q), an inhibitor of the human CDK9 was extracted from the complex and saved in sdf format. All ligand structures were then energy minimized using the universal force field (UFF) under the Conjugate Gradient algorithm in 200 steps before being converted to the partial charge and atom type (pdbqt) file format of the Protein Data Bank using Open Babel.

Both *Ld*CRK12 and the human CDK9 were energy minimized using the Optimized Potentials for Liquid Simulations (OPLS)/All Atom (AA) force field in GROMACS 2018. PyMOL (PyMOL Molecular Graphics System, Version 1.5.0.4, Schrödinger, LLC) was used to visualize the energy minimized structures and to remove the water molecules surrounding the protein. The protein structures were then saved in the Protein Data Bank format (pdb) using PyMOL. The protein structures were then converted to AutoDock Vina’s compatible pdbqt format using the “make macromolecule” option in PyRx.

### 2.6. Virtual Screening

Autodock Vina was employed for the virtual screening process [54]. The pre-filtered library and the known drugs were screened against the *Ld*CRK12 using a grid box dimension of 91.21 × 93.45 × 78.24 Å^3^ and centered at (74.47, 128.44, 81.76) Å to cover the kinase domain. Compounds that possessed binding energies higher than −8.5 kcal/mol were not selected. A more stringent threshold was used herein since a previous study showed that −7.0 kcal/mol which was defined for AutoDock users can significantly distinguish between putative specific and non-specific protein–ligand bonds [55]. The result was then inspected visually using PyMOL to select the best docked ligands. 

The known ligands and the selected compounds were re-docked to the human CDK9 using AutoDock Vina. The CDK9 protein was remodelled using the existing CDK9 structure (PDB ID: 4BCF) as a template via Modeller before molecular docking studies due to missing residues. A grid box with the dimension of 80.86 × 62.73 × 91.07 Å^3^ and center (81.89, 80.83, 70.34) Å was specified for the CDK9. Compounds that demonstrated a higher binding affinity to the human CDK9 than 2-amino-4-heteroaryl-pyrimidine were not considered for downstream analysis.

### 2.7. Characterisation of Mechanism of Binding

The interactions between *Ld*CRK12 and the ligands were determined and analyzed via LigPlot + v1.4.5 using default parameters [56]. Additionally, the human CDK9–ligand interactions were investigated.

### 2.8. Pharmacological Profiling

Selected compounds with high binding affinities with the *Ld*CRK12 protein and low binding affinities to the human CDK9 were subjected to absorption, distribution, metabolism, and excretion (ADME) evaluation using SwissADME [57]. The toxicity profiles of the selected compounds were evaluated using OSIRIS Property Explorer in DataWarrior 5.0.0 [58]. DataWarrior uses features of chemical structures to predict physicochemical properties. The algorithm in the OSIRIS Property Explorer predicts the likelihood of a drug being a mutagenic, tumorigenic, irritant, and possessing a reproductive effect. Prediction of activity spectra for substances (PASS) was used to predict the biological activity of the compounds. PASS predicts the biological activity spectra of compounds using the simplified molecular input line entry system (SMILES) files of the structures based on the Bayesian approach [27,28].

### 2.9. Quality Evaluation of Shortlisted Molecules

The inhibitory constant (Ki) was calculated using the binding energies of the selected compounds along with other metrics consisting of ligand efficiency (LE), LE scale (LE_Scale), fit quality (FQ), and LE-dependent lipophilicity (LELP). The abovementioned metrics were determined using the method described previously [59,60].

### 2.10. MD Simulations of Proteins and Protein–Ligand Complexes

A 10 ns MD simulation was performed for *Ld*CRK12 and protein–ligand complexes using GROMACS 2018 [61,62]. Xmgrace [63] was used to plot the graphs generated from the MD simulations. The binding free energies of the complexes were calculated using the MM/PBSA method [25]. MM/PBSA calculations of the complexes were carried out using g_MM/PBSA, which calculates binding energy components and the individual energy contributions of the residues [25]. The graphs from the MM/PBSA computations were generated using the R programming package [64].

## 3. Results and Discussion

The results of the molecular modelling, molecular docking, ADMET evaluation, prediction of antileishmanial activity and MD simulations are presented.

### 3.1. Modelling the Structure of LdCRK12

There was the need to model the structure of the *Ld*CRK12 since there is no available structure in the protein data bank. An earlier study modelled the structure of the *Ld*CRK12 using Molecular Operating Environment (MOE version 2014.09; Chemical Computing Group, Inc.) [20]. MOE-Homology combines segment-matching and methods of inserting or deleting regions to model protein structures. Advanced knowledge-based loop searching and sidechain rotamer selection methods are then employed to build models by default. An average model is then generated by MOE for a user-controlled energy minimization [65]. 

Studies have compared the quality of protein structures generated using different modelling techniques [65,66,67]. No technique has been found to be superior in every aspect to the others [65,66]. The protein family and the sequence identity between the query and template structures influence the quality of a model built using a homology modelling technique [66]. A comparison study of various homology modelling algorithms including MOE, I-TASSER, Rosetta, PRIME, SWISS-MODEL, Composer, and ORCHESTRAR reported that all the techniques produced high quality models when the sequence identity between the query and the template is greater than 35% [66,67]. However, for low sequence identities, it becomes difficult for the modelling algorithms to produce high-quality structures [66]. It is therefore imperative that different modelling techniques are used to build protein structures that have relatively low sequence identities to their templates. The quality of the modelled structures must be assessed to select the reasonably best model.

Herein, three freely accessible and widely used techniques comprising Modeller, I-TASSER and Robetta were employed to predict structures of the *Ld*CRK12. The present study compares the structures from these three techniques to select the reasonably best model, as carried out previously [68,69,70].

#### 3.1.1. Template Search

A BLAST search was performed to retrieve identical structures as suitable templates for modelling the *Ld*CRK12 structure. The BLAST search via SWISS-MODEL revealed 5449 templates with a sequence identity lower than 30%. A further BLAST search was conducted on the kinase domain (amino acid residues 459–833) using the BLAST option (BLASTP 2.9.0+) by selecting BLOSUM62, the most commonly used scoring matrix in BLAST [71]. The search revealed six reviewed protein structures that were identical to the kinase domain of the *Ld*CRK12 (Table 1). One of the most widely used template selection criteria is to select the model with the highest sequence identity to the protein sequence. The quality of the experimentally determined structure is also an important factor to consider in the template selection. The reasonably best template was selected based on the E-value, sequence identity, query coverage, and the availability of a 3D structure. The human CDK9 was thus selected as the template for modelling the *Ld*CRK12 via Modeller 9.2 as described previously [20]. Although, sequences O14098 and Q9TVL3-2 had sequence identities of 36% and 35% and BLAST scores of 356 and 348, respectively, they were not selected due to their relatively low coverage to the *Ld*CRK12 (Table 1). Cyclin-dependent kinase 9 (CDK9) of humans, rats, and mice had the same E-value, BLAST score, and sequence identity of 7.4 × 10^−34^, 345, and 31.3%, respectively. The three proteins also had better coverage of the *Ld*CRK12. However, the human CDK9 was the only protein with a solved 3D structure.

#### 3.1.2. Structure Prediction Using Modeller

Modeller 9.2 was employed to generate five structures of the *Ld*CRK12 using the human CDK9 (PDB ID: 4BCF) as a suitable template [20]. The human cyclin-dependent kinase 9 (CDK9) is a cdc2-like serine/threonine kinase whose related pathways have been associated with various human malignancies and cardiomyocyte hypertrophy. The sequence of the *Ld*CDK12 was aligned to the template sequence and five structures were modelled using Modeller 9.2.

The qualities of the five generated models were evaluated using the DOPE and genetic algorithm 341 (GA341) scores. The GA341 score, which is derived from statistical potential, assesses the reliability of a model [72]. A model can be said to be reliable when the GA341 score is higher than the determined threshold of 0.7. The five generated models using Modeller 9.2 had a GA341 score lower than the 0.7 cut-off, thus the DOPE score was used to select the most suitable model. The DOPE score is also a statistical potential score used to assess predicted models. The reasonably best model is selected by choosing the structure with the least DOPE value [30,43]. The DOPE and GA341 scores of the five predicted models from Modeller 9.2 are shown (Table 2). For the Modeller generated structures, model MOD5 was selected as the most suitable structure of the *Ld*CRK12 due to its very low DOPE score of −50486.88281 (Table 2 and Supplementary file 1).

#### 3.1.3. Structure Prediction Using I-TASSER

I-TASSER was used to generate five protein structures of the *Ld*CRK12. Based on the magnitude regarding the threading template alignments and the convergence parameters of the structure assembly simulations, I-TASSER computed a confidence rating for each model, which is known as the C-score. A higher C-score value represents a model with higher confidence and is usually in the range of (−5, 2) [31,32,33,34]. Out of the five generated I-TASSER structures, model ITAS5 was selected as the most suitable model due to its high C-score of −2.66 (Table 3 and Supplementary file 2).

#### 3.1.4. Structure Prediction Using Robetta

Robetta was also employed to model five structures of the *Ld*CRK12. Robetta uses the ROSETTA to model protein structures either by comparative modelling or ab initio. For the *Ld*CRK12, Robetta used comparative modelling to predict plausible structures (Table 4). ROB1 was considered as the reasonably best model since the predicted models are ranked based on the model quality assessment method available in ProQ2 after clustering. The predicted b-factors by color representation of the models were also visualized in Pymol. The b-factor, which influences the local quality of a model, shows the parts of the structure that were remodelled and not covered by a template. These regions are the least accurate and have the most variation between models. All five predicted structures showed similar b-factor coloration. Therefore, the five models were further evaluated using SAVES v5.0 (Table 4). ROB1 had a VERIFY score of 82.97%, which was the highest; ERRAT quality factor of 88.0579; PROVE score of 0.0% and four PROCHECK errors, three warnings, and two passes (Table 4). ROB1 was thus selected as the most acceptable structure from Robetta (Supplementary file 3).

### 3.2. Quality Assessment of Selected Models

**Figure 2:**
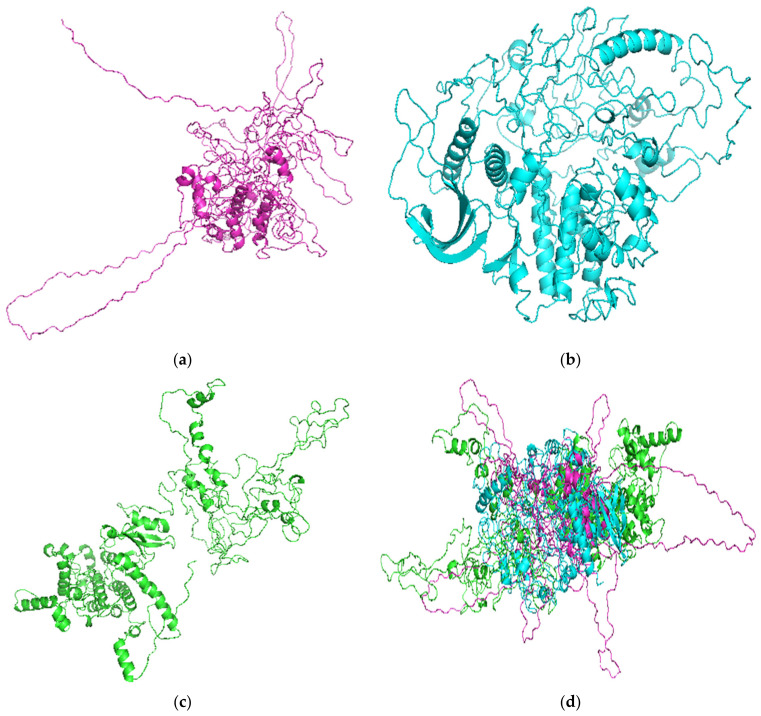
Cartoon views of the top 3 predicted tertiary structures of the *Ld*CRK12 from the 3 techniques used: (**a**) MOD5; (**b**) ITAS5; (**c**) ROB1; and (**d**) top 3 models aligned. MOD5, ITAS5, and ROB1 are colored in magenta, cyan and green, respectively.

The quality of the best models from each of the three techniques was assessed using SAVES v5.0. Modelled protein structure MOD5 had poor values for all the quality metrics (Table 5). MOD5 had VERIFY, ERRAT, and PROVE scores of 41.20%, 10.0536, and 16.1%, respectively. MOD5 was also predicted by PROCHECK to have five errors, two warnings, and one pass (Table 5). ITAS5 had very good VERIFY and ERRAT scores of 85.36% and 80.2158, respectively. Although ITAS5 had the highest VERIFY score, it was predicted using PROVE to be 9.5% erroneous (Table 5). PROCHECK also predicted ITAS5 to have six errors, two warnings, and one pass. ROB1 had the highest ERRAT quality factor of 88.0579 and 0.0% erroneous parts, as predicted by PROVE (Table 5). The ERRAT error plots for MOD5, ITAS5, and ROB1 were generated (Figure S1). MOD5 had the most erroneous or misfolded regions (Figure 2a and Figure S1A), while ROB1 had the lowest error rate for protein folding (Figure 2c and Figure S1C). Furthermore, the kinase domain of the *Ld*CRK12 (residues 459–833) in the ROB1 structure was not predicted to have any misfolded or erroneous regions (Figure S1C). ITAS5 was also observed to have few misfolded portions (Figure 2b and Figure S1B).

**Figure 3:**
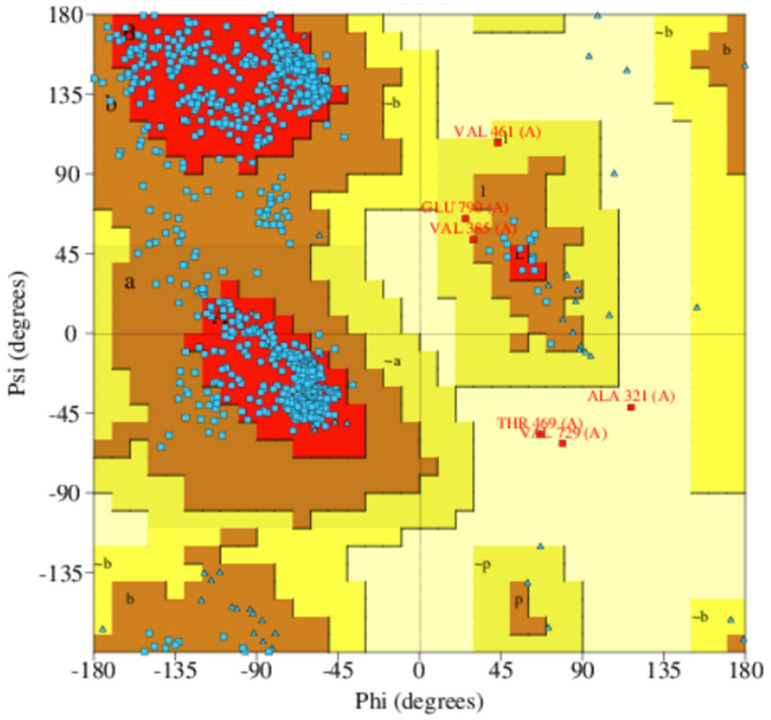
Ramachandran plot of the selected *Ld*CRK12 structure (ROB1) obtained via PROCHECK. The percentages of residues in the most favored regions, additionally allowed regions, generously allowed regions and disallowed regions are 82.0%, 17.1%, 0.4%, and 0.4%, respectively.

The Ramachandran plots of all the three shortlisted models were obtained using PROCHECK which evaluates the stereochemistry of protein models by determining residue-by-residue geometry and overall structure geometry [44]. A protein structure is considered as quality based on the percentage of residues in the most favored (core), additionally allowed, generously allowed, and disallowed regions [73]. Protein structure MOD5 had 79.7%, 15.5%, 3.0%, and 1.8% of residues in the most favored, additionally allowed, generously allowed, and disallowed regions, respectively (Table 6 and Figure S2A). ITAS5 was also predicted to have 61.0%, 29.8%, 5.9%, and 3.3% of residues in the most favored, additionally allowed, generously allowed, and disallowed regions, respectively (Table 6 and Figure S2B). For the ROB1 structure, 82% of the amino acid residues were present in the most favored region, 17.1% residues were found in the additionally allowed regions, 0.4% of residues were in the generously allowed regions, and 0.4% in the disallowed region (Table 6 and Figure 3). The Ramachandran plots revealed that the model ROB1 had the most reasonably good structure (Table 6, and Figure 3 and Figure S2A,B).

**Figure 4:**
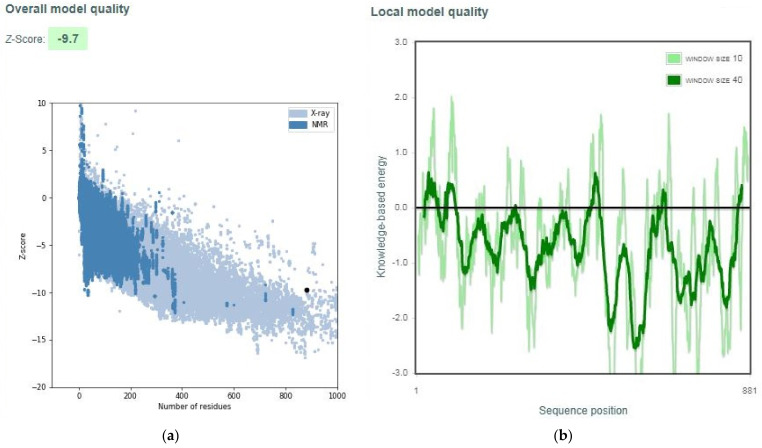
Model quality assessment using ProSA-web. (**a**) A z-score of the best *Ld*CRK12 structure indicating the overall model quality, and (**b**) a local model quality of the selected *Ld*CRK12 structure by plotting energies as a function of amino acid sequence position.

The quality of the overall best model (ROB1) was evaluated using the z-score from ProSA-web [45,46]. The overall best model was predicted to be of X-ray quality and had a z-score of −9.7 (Figure 4a). The local model quality of the chosen model was also determined by plotting the energies as a function of amino acid residue position. Most of the residues were predicted to have negative energy values, signifying a very good model (Figure 4b). Generally, positive values signify problematic or erroneous parts of the input structure.

### 3.3. Binding Site Characterization

A binding site is a region on a protein that binds to a ligand or another macromolecule with specificity [74]. CASTp was employed to predict the binding sites of the *Ld*CRK12. At the active site, a ligand or a substrate binds to an enzyme to induce a chemical reaction [75]. CASTp uses the Delaunay triangulation, alpha shape, and discrete flow methods to identify topographic features, measure areas and volumes [76,77]. 

**Figure 5:**
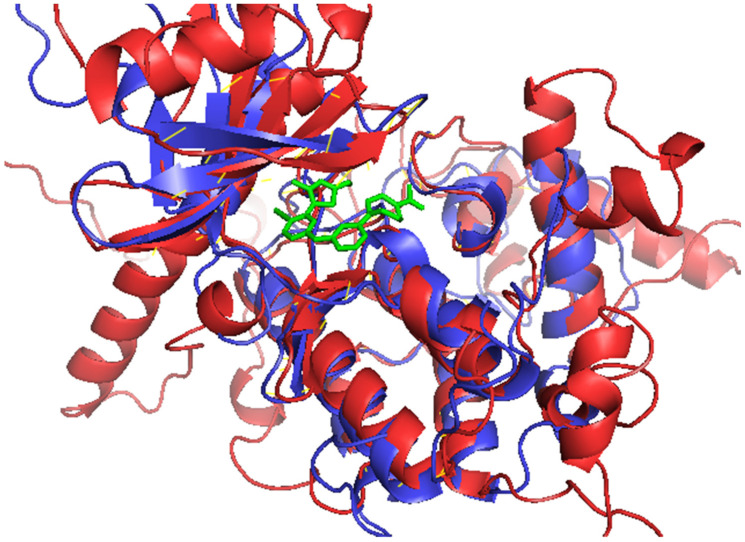
Superimposition of human CDK9-T6Q complex with the *Ld*CRK12. *Ld*CRK12, CDK9, and T6Q are colored in red, blue, and green, respectively.

CASTp predicted 127 binding sites for the chosen *Ld*CRK12 protein model. The predicted binding cavities with no openings and with relatively small volumes and areas such that no ligand could fit were ignored [59,78]. Since the modelled structure had many disordered regions from residues Met1 to about Ala400, only binding cavities predicted to border the kinase domain (459–833) were considered. A total of 14 binding sites were selected after visualization in Chimera 1.12 and Pymol. The residues lining each of the 14 binding sites are shown (Table 7). Pocket 7 was observed to overlap with pocket 3 (Table 7). Aligning the 3D structures of the *Ld*CRK12 and the human CDK9 in complex with T6Q revealed that pocket 1 is the ATP binding site of the kinase domain (Figure 5 and Table 7).

### 3.4. Preparation of Screening Library

A total of 7722 African natural compounds were used as the screening library [52,53]. Additionally, Lipinski’s rule of five was used to filter the library to obtain 4409 compounds comprising 3872 and 537 ligands from the NANPDB and AfroDB, respectively. 

Three known antileishmanial drugs, namely amphotericin B, miltefosine, and paromomycin, were also retrieved from PubChem with CIDs 5,280,965, 3599, and 165,580, respectively. Three inhibitors of *Ld*CRK12 comprising compounds 5, 7, and 8, which had very good EC_50_ values in mouse models of visceral leishmaniasis ranging from 0.005 µM to 2 µM, were also used. The 3D structure of GSK3186899 was downloaded from PubChem with CID 122,429,808 whereas those of compounds 5 and 8 were generated using MarvinSketch 17.17.0. Additionally, a 2-amino-4-heteroaryl-pyrimidine inhibitor (Code: T6Q), complexed with the human CDK9 (PDB ID: 4BCF) was extracted from the complex and saved in sdf format.

All ligand structures were energy minimized using the universal force field (UFF) under the Conjugate Gradient algorithm in 200 steps and converted to the partial charge and atom type (pdbqt) format using Open Babel before the virtual screening.

### 3.5. Virtual Screening of Compounds

Autodock Vina was used for the virtual screening process [54]. The compounds were first screened against the *Ld*CRK12. Compounds with good pose and low binding energies against the *Ld*CRK12 were re-docked against the human CDK9 to select compounds that are less likely to interact with critical residues of the human CDK9.

#### 3.5.1. Screening the Library against *Ld*CRK12

The pre-filtered library comprising a total of 4409 compounds and the known inhibitors were screened against the energy minimized *Ld*CRK12 using a grid box dimension of 91.21 × 93.45 × 78.24 Å^3^ and centered at (74.47, 128.44, 81.76) Å to cover the kinase domain of the protein. A total of 4369 compounds were successfully screened against the *Ld*CRK12. A stringent threshold of −8.5 kcal/mol was used to select the compounds after the virtual screening process. This threshold was used since it has been shown that an AutoDock score of −7.0 kcal/mol differentiates well between certain and uncertain protein–ligand interactions [55]. A total of 290 compounds had binding energies less than or equal to −8.5 kcal/mol. AutoDock Vina uses a negative function to rank the output in the order of decreasing binding affinity, thus, the higher the negativity, the more plausible the candidate as a potential lead compound. 

The protein–ligand complexes were then inspected visually using PyMOL to select the best docked ligands. A total of 17 compounds were eliminated since they did not dock deep into the *Ld*CRK12. Additionally, based on the generated protein–ligand interaction profiles, 27 compounds that did not exhibit any hydrogen bonding with *Ld*CRK12 were excluded. A total of 246 compounds were thus selected from the virtual screening output. Of the 246 compounds, ZINC000095485940 demonstrated the least binding energy to the *Ld*CRK12 with a value of −10.1 kcal/mol. NANPDB1406, NANPDB2581 and NANPDB6446 also demonstrated low binding energies of −9.5, −9.2 and −9.1 kcal/mol, respectively. ZINC000095485940, NANPDB1406, NANPDB2581, and NANPDB6446 demonstrated a higher binding affinity to the *Ld*CRK12 than all the known inhibitors used in this study (Table 8).

Among the known inhibitors, the compound 8 and TQ6 demonstrated the least binding energy of −9.1 kcal/mol to the *Ld*CRK12. Compound 8 was reported to inhibit the *Leishmania* parasite with EC_50_ values of 0.025 µM and 0.075 µM in the axenic and intra-macrophage assays, respectively. GSK3186899, paromomycin, and compound 5 also had binding energies of −8.5, −7.9, and −7.2 kcal/mol, respectively (Table 8). These three compounds demonstrated binding energies lower than the −7.0 kcal/mol threshold defined for AutoDock users [55]. This implies that these compounds have the potential to demonstrate significant inhibitory activities against the parasite as exhibited by compounds 5 and 7 previously [20]. Miltefosine demonstrated the highest binding energy of −5.0 kcal/mol to the *Ld*CRK12 (Table S1).

#### 3.5.2. Re-Docking Compounds against the CDK9

Since the kinase domain is conserved and the human CDK9 is homologous to the *Ld*CRK12, there was the need to screen the shortlisted compounds against the CDK9. A total of 246 were re-docked against the human CDK9 to select compounds with a relatively low binding affinity to the CDK9, which were less likely to interact with critical residues of the human CDK9. Before the virtual screening, the CDK9 was remodelled with PDB structure 4BCF as a template using Modeller 9.2 due to missing residues. Residues 1–5, 89–96, 177–181, and 327–330 were missing in the human CDK9 structure. The complete sequence of the human CDK9 was retrieved from UniProt with ID P50750 [38,39,40]. The sequence was aligned to the 4BCF structure and five models were generated using Modeller 9.2. The qualities of the five models were assessed using the DOPE and GA341 scores. All the modelled structures had a GA341 score of 1, thus the structure with the lowest DOPE (−38809.11328) score was chosen.

Ligands that docked into the ATP binding site of the human CDK9 were not considered for downstream analysis. Additionally, compounds with similar binding energy against the CDK9 as T6Q were eliminated to prevent the likelihood of drug off-target binding. T6Q had a binding energy of −8.6 kcal/mol when docked into the ATP binding site of the human CDK9 (Table 8). 

Compounds 8, GSK3186899, and 5 were observed to have binding energies of −9.0, −8.8 and −8.6 kcal/mol against CDK9, respectively (Table 8). However, GSK3186899 had an IC_50_ value higher than 20 μM against the human CDK9 [20]. Miltefosine had the lowest binding affinity to CDK9, with a binding energy of −5.6 kcal/mol (Table 8). 

ZINC000095486260 demonstrated the highest binding energy (−6.4 kcal/mol) against CDK9, followed by NANPDB4609 and NANPDB328, with both having a binding energy of −6.6 kcal/mol each (Table 8). ZINC000095485940, NANPDB1406, NANPDB2581, and NANPDB6446, which had the highest binding affinity to *Ld*CRK12, had binding energies of −7.7, −7.3, −7.5 and −7.3 kcal/mol with the human CDK9, respectively (Table 8). A total of 133 compounds with a high binding affinity against the CDK9 were eliminated.

### 3.6. Characterisation of Mechanisms of Binding

The protein–ligand interactions were determined for both *Ld*CRK12- and the human CDK9–ligand complexes using LigPlot + v1.4.5 [56].

#### 3.6.1. Characterization of *Ld*CRK12–Ligand Interactions

Most compounds were observed to dock into the ATP binding pocket, consistent with pocket 1 (Table 7; Table 8) with paromomycin and T6Q docking into the ATP binding cavity. Compounds 5 and 8 docked into pocket 14 and formed hydrogen bonds with Leu723 (Table 7; Table 8). Compound 5 formed 2 hydrogen bonds with Leu723 of lengths 2.98 and 3.07 Å, and interacted with Gly724, Pro725, Leu726, Pro727, Pro728, Val731, Tyr732, Leu743, Asn763, Trp764, Gln815, Leu816, Asp817, and Gln820 via hydrophobic bonds. Compound 8, which had a binding affinity of −9.1 kcal/mol with the *Ld*CRK12 interacted via one hydrogen bond with Leu723 (2.83 Å), and formed hydrophobic contacts with Gly724, Pro725, Leu726, Pro727, Pro728, Val731, Leu743, Glu747, Asn763, Trp764, Gln815, and Leu816. The interactions between compounds 5 and 8 with these residues may account for their high *L. donovani* inhibitory activity.

**Figure 6:**
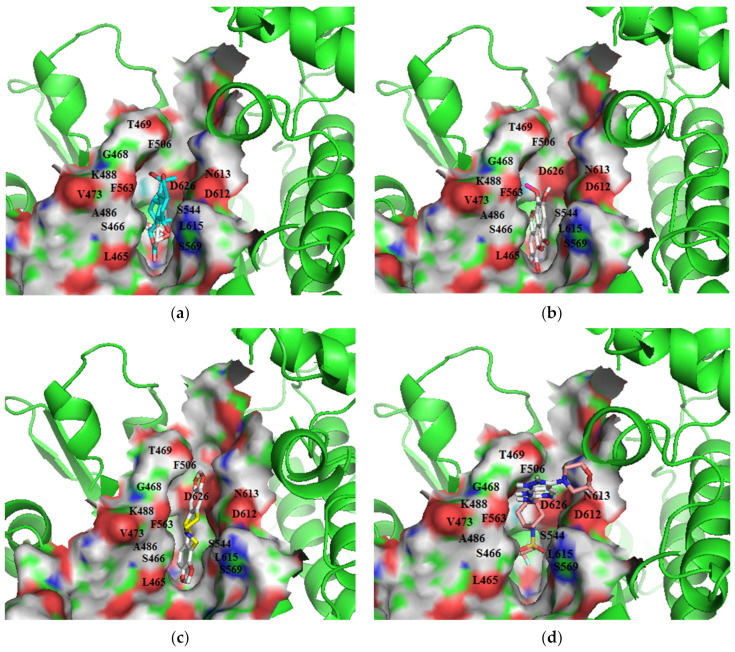
Cartoon representation of *Ld*CRK12 in complex with: (**a**) ZINC000095485940; (**b**) NANPDB1406 (methyl ellagic acid); (**c**) NANPDB2581 (stylopine); and (**d**) GSK3186899 (Compound 7). The binding site is shown as surface representation with the ligands shown as sticks.

GSK3186899, which docked into pocket 1, interacted with Ser466 (2.96 Å), Gly468 (3.19 Å), Lys488 (3.03 Å), Ser544 (3.27 Å), Thr625 (3.12 Å), Asp626 (3.31 Å, 3.3 Å), and Tyr691 (2.98 Å) via hydrogen bonding, and Gly468, Thr469, Tyr470, Val473, Ala486, Lys488, Phe563, Lys610, Asp612, Leu615, Asp626, and Tyr691 via hydrophobic bonding (Figure 6d and Figure S3D, and Table 7; Table 8). The multiple hydrogen bonding formed between GSK3186899 and the *Ld*CRK12 may be a key influencer of its activity [79].

ZINC000095485940 interacted with the *Ld*CRK12 via hydrogen bonds with Gly468, Ser569 and Asp626 of bond lengths 2.93, 2.95 and 2.70 Å, respectively (Figure 6a and Figure S3A, and Table 8). ZINC000095485940 also formed hydrophobic contacts with Leu465, Ser466, Thr469, Val473, Ala486, Lys488, Ser544, Phe563, Asp612, Asn613, Leu615, and Thr625 (Figure 6a and Figure S3A, and Table 8). NANPDB1406 interacted with Lys488, Ala566, and Ser569 via hydrogen bonds and also formed hydrophobic bonds with Leu465, Ser466, Gly468, Val473, Ala486, Tyr565, Thr567, Ala568, Asp612, Leu615, and Asp626 (Figure 6b and Figure S3B, and Table 8). NANPDB2581 formed a hydrogen bond with Lys610 with a bond length of 3.08 Å and hydrophobic contacts with Leu465, Ser466, Thr469, Tyr470, Ala568, Ser569, Asp612, Asn613, Leu615, and Asp626 (Figure 6c and Figure S3C, and Table 8). NANPDB6446 also interacted with the *Ld*CRK12 via hydrogen bonds with Ser569 and Arg575, and hydrophobic bonds with Leu465, Ser466, Ala568, Gly572, Asp612, and Asp626. The formation of multiple hydrogen bonds between an enzyme and a molecule influences the activity of the compound [79]. Leu465, Ser466, Thr469, Ala486, Ala568, Ser569, Asp612, Asn613, Leu615, and Asp626 are predicted as critical residues for ligand binding in the ATP binding pocket.

Amphotericin B docked into binding pocket 3 forming hydrogen bonds with Arg603, Pro635, Tyr845, Gln846, and Arg847 (Table S1). Amphotericin B also interacted with Pro635, Gly636, Thr642, His643, Glu669, Lys670, Thr823, Glu826, Tyr845, Gln846, Arg847, and Leu849 via hydrophobic contacts. These residues were found to line binding pocket 3 (Table 7). NANPDB2521 and NANPDB1011 also formed interactions with the aforementioned residues. 

Miltefosine formed 2 hydrogen bonds with Gly422 with bond lengths of 3.05 and 3.1 Å, and interacted with Leu181, Gly344, Ile345, Thr396, Arg397, Ala399, Pro401, Thr418, Pro419, Tyr420, Pro421, Gly422, Tyr428, and Arg432 via hydrophobic bonds (Table 8), which lined pocket 9 (Table 7). Pockets 3 and 9 are worthy of further experimental exploration.

#### 3.6.2. Characterization of Human CDK9–Ligand Interactions

The human CDK9–ligand interactions were also investigated (Table 8 and Table S1). Ligands which interacted with the critical residues of the human CDK9 (Ile25, Ala46, Lys48, Phe103, Glu107, Asp109, Asp145, Leu156, and Asp167) were not considered for downstream analysis due to the possibility of drug off-target activity [80,81]. Previous studies have reported on the crystal structures of analogues of 4-(thiazol-5-yl)-2-(phenylamino)pyrimidine-5-carbonitrile bound to CDK9/cyclin T [82,83]. The compounds demonstrated Ki values ranging from 6–43 nM with an increase in the thermal stability of CDK9/cyclin T [82]. It was reported that the thiazole, pyrimidine, and aniline moieties docked into the ATP binding site and formed a hydrogen bond with the hinge region of the kinase [82]. The pyrimidine ring was observed to lie between Ala46 and Leu156 while the C5-carbonitrile was reported to form a lone pair−π interaction with an average distance of 3.7 Å with the gatekeeper residue Phe103 [82]. Hydrogen-bonds were also formed between the compounds and residues Ile25, Lys48, Asp145, with Glu107 and Asp167 [82]. Other studies have corroborated the above listed residues as being critical to CDK9–ligand binding [84,85]. A molecular docking study involving CDK9 and BAY-958 also reported BAY-958 to form a hydrogen-bond with Asp109 [84].

A total of 94 compounds that interacted with the critical residues of the human CDK9 were eliminated from this study. A total of 19 compounds with a relatively high binding affinity to the *Ld*CRK12 and did not interact with the critical residues of the human CDK9 were obtained.

### 3.7. ADMET Prediction

Though the screening library was pre-filtered using Lipinski’s rule, Veber’s rule was further applied to the 19 identified compounds, of which two failed. NANPDB4609 and NANPDB3239 violated Veber’s rule due to their high total polar surface area (TPSA) values of 151.96 and 145.91, respectively. Veber’s rule requires a TPSA of no more than 140 Å^2^ [86]. Compounds with a TPSA not more than 140 Å^2^ are considered to have good oral bioavailability [86]. TPSA values are considered as good indicators of excellent human intestinal absorption (HIA) and Caco-2 permeability [87]. The calculated logP (cLogP) values were also determined using the OSIRIS DataWarrior 5.0.0 (Table S2). 

Most of the compounds were predicted to be moderately soluble, including compounds 5, 7, and 8 (Table S2). Compound 5 was shown experimentally to have poor solubility and is metabolically unstable although it was the most potent against *Ld*CRK12 with an EC_50_ value of 0.014 μM in the intra-macrophage assay [20]. NANPDB6446 was predicted to be very soluble while NANPDB1406 was predicted to be moderately soluble. ZINC95485940, NANPDB1406, and NANPDB1649 were also predicted to be soluble (Table S2). 

The potential of a drug to move across the blood–brain barrier to the brain is referred to as BBB permeation. Only NANPDB2581, NANPDB2582, NANPDB3614, NANPDB1649 and ZINC000095485880 were predicted to have permeation into the brain–blood barrier (BBB) [Table S2]. In the brain, the drug binds to specific receptors to activate certain signaling pathways. Additionally, for a drug to exhibit the desired pharmacological activities with the brain parenchyma, it needs to be able to permeate the BBB [88].

T6Q, compound GSK3186899, compound 5, NANPDB4609, NANPDB3239, amphotericin B, paromomycin, and miltefosine were predicted to have low gastrointestinal (GI) absorption, which suggests a low probability of successful absorption into the bloodstream (Table S2). Another factor considered was the likelihood of the compounds to be non-P-glycoprotein (P-gp) substrates. P-gp aids in the removal of drugs or xenobiotics from the central nervous system (CNS) by functioning as a biological barrier by removing toxins and xenobiotics from cells. It is also crucial in the absorption and distribution of drugs [89]. All the inhibitors or drugs used in this study were predicted to be P-gp substrates (Table S2). Of the top 19 hits, 10 compounds were predicted to be non-P-gp substrates (Table S2) and may likely have desirable distribution in the circulatory system upon administration.

### 3.8. Toxicity Prediction with OSIRIS Property Explorer

The toxicity profiles of the 17 hits and the known drugs were determined using OSIRIS DataWarrior 5.0.0 (Table S3). Of the 17 hits, 13 compounds were predicted to be non-tumorigenic, non-mutagenic and non-irritant, and to have no reproductive effects (Table S3). NANPDB6446 was predicted to be highly mutagenic, tumorigenic, and irritant. NANPDB6446 can serve as a scaffold for fragment-based drug design due to its relatively low molecular weight of 365.381 g/mol.

NANPDB3614 and ZINC000000828203 were also predicted to be highly tumorigenic while NANPDB3284 was predicted to have reproductive effects (Table S3). Compounds 5, 8, amphotericin B, miltefosine, paromomycin, and T6Q were also predicted to have no mutagenicity, tumorigenicity, irritancy, and reproductive effect (Table S3). GSK3186899 was predicted to possess low tumorigenicity though it was non-mutagenic, non-irritant, and had no reproductive effect (Table S3). GSK3186899 was selected as the preclinical candidate due to its effectiveness, efficacy, pharmacokinetics, and safety profile [20]. GSK3186899 was reported to possess *L. donovani* inhibitory activity in cidal axenic amastigote and intra-macrophage assays with EC_50_ values of 0.1 and 1.4 μM, respectively [20]. 

### 3.9. Biological Activities of Hits

The biological activities of the 17 identified hits were determined using PASS, an Open Bayesian machine learning technique. Structure descriptors, which are also referred to as multilevel neighborhoods of atoms (MNAs) descriptors, were generated as inputs [27].

A total of 13 compounds were predicted to possess antiprotozoal activity, of which 10 were predicted to be antileishmanial (Table S4). NANPDB1406, NANPDB2521, NANPDB3435, NANPDB3284 and ZINC000095486260 were predicted as kinase inhibitors (Table S4). Since the *Ld*CRK12 has a kinase domain, these predictions necessitate the in vitro testing of these compounds to validate their anti-*Ld*CRK12 and antileishmanial properties. Fifteen of the hits were predicted to possess antineoplastic (anticancer) activity (Table S4). A review on the in vitro leishmanicidal potential of anticancer compounds suggested the use of antineoplastic compounds for the treatment of leishmaniasis [90]. Protein kinase inhibitors such as sunitinib, sorafenib, and lapatinib which are used for treating cancers were reported to be active against *Leishmania donovani* amastigotes in murine macrophages with IC_50_ values of 1.1, 3.7, and 2.5 μM, respectively, showing similar efficacy to that of miltefosine (IC_50_ = 1.0 μM) [91]. Sunitinib, sorafenib, and lapatinib were also reported to be non-toxic to mammalian cells [91]. 

NANPDB1011, NANPDB3949, ZINC000095486260, NANPDB3435, NANPDB3284 and NANPDB2521 were predicted to possess dermatological activities. These compounds may be beneficial in treating post kala-azar dermal leishmaniasis (PKADL). NANPDB1649 (sesamin) has been reported to be active against *Leishmania amazonensis* with an IC_50_ value of 15.8 µg/mL and was not cytotoxic to macrophage cells with CC_50_ values greater than 100 µg/mL [92]. Additionally, ZINC000000828203 (diphyllin) isolated from *Haplophyllum bucharicum* (Rutaceae) has been reported to demonstrate antileishmanial activity against *Leishmania infantum* promastigotes and intracellular amastigotes with IC_50_ values of 14.4 μM and 0.2 μM, respectively [93]. NANPDB3614 (justicidin B) has also been shown to be a potential antiprotozoal agent by showing antitrypanosomal activities against *Trypanosoma brucei rhodesiense* and *Trypanosoma cruzi* with IC_50_ values of 0.2 and 2.6 µg/mL, respectively [94]. Since *Leishmania* and *Trypanosoma* are trypanosomatids, repurposing NANPDB3614 for the development of therapeutic agents for leishmaniasis can be explored.

### 3.10. Ligand Efficiency-Based Metrics for Selected Compounds

Quality metrics for the top compounds such as the inhibitory constant (Ki), ligand efficiency (LE), fit quality (FQ), LE scale (LE_scale), and LE-dependent lipophilicity (LELP) were determined as described previously [59,60]. The predicted Ki values ranged from 0.039 to 0.587 μM (Table 9). ZINC000095485940 demonstrated the lowest predicted Ki value of 0.039 μM while NANPDB1649 (sesamin) showed the highest Ki value of 0.587 μM against the *Ld*CRK12 (Table 9; Table 10). Sesamin was shown to inhibit *L. amazonensis* with an IC_50_ value of 15.8 µg/mL (44.588 μM) [92]. The relatively low Ki values indicate the potential inhibitory activities of the selected compounds [95].

The ligand efficiency (LE) of the selected compounds ranged from 0.327 to 0.413 (Table 9) which are very close to the average ligand efficiency values reported for fragment-like compounds (0.38). LE is used to assess the binding affinity, taking into account the number of heavy atoms (NHA) of a molecule [96,97]. Herein, NANPDB1649 demonstrated the lowest LE value of 0.327. ZINC000095485940, NANPDB6446, NANPDB2581 and NANPDB1406 had LE values of 0.347, 0.380, 0.404 and 0.416, respectively (Table 9; Table 10). Similarly, these LE values are close to the average LE values of fragment-like molecules (0.38) [97].

The LE_Scale takes into consideration size dependency, which is a limitation of the LE metric. The computed LE_Scale values ranged from 0.347 to 0.416 (Table 9), in concordance with the LE_Scale values of similar active compounds with the same number of heavy atoms [98,99]. ZINC000095485940 had the lowest LE_Scale value of 0.347, while NANPDB1406 had the highest value of 0.416. NANPDB2581, NANPDB6446 and NANPDB1649 also had LE_Scale values of 0.404, 0.380 and 0.380, respectively (Table 9).

The fit quality (FQ), which is a more accurate metric used to assess ligand efficiency, is determined as a ratio of the observed LE to the LE_Scale of a compound. The closer the FQ to 1, the more ideal the ligand. The calculated FQ values ranged from 0.861 to 1.003 (Table 9), suggestive that the selected molecules have plausible binding to the *Ld*CRK12 receptor [97].

Another important metric, ligand-efficiency-dependent lipophilicity (LELP) was also computed for the selected molecules. For a promising compound, the recommended LELP should be between 0 and 7.5, although molecules that satisfy Lipinski’s rule are reported to have LELP values less than 16.5 [100]. The LELP values of all proposed molecules ranged between 0.521 and 9.861, which suggests that the selected molecules have a good affinity to *Ld*CRK12, considering lipophilicity. ZINC000095485940, NANPDB1406 and NANPDB6446 had LELP values of 0.521, 3.761 and 2.370, respectively (Table 9).

### 3.11. Molecular Dynamics Simulations

**Figure 7:**
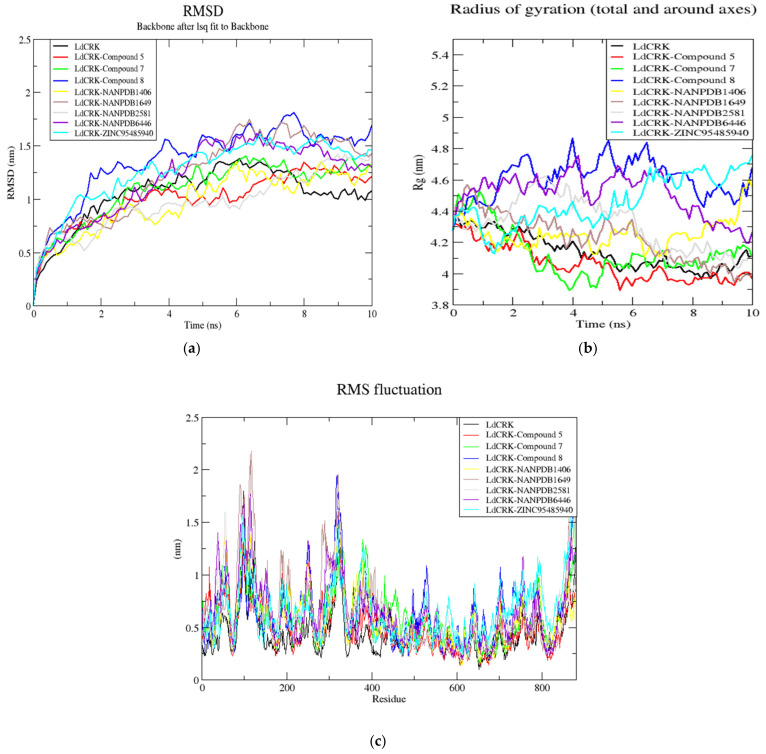
The root mean square deviation (RMSD), radius of gyration (Rg), and root mean square fluctuation (RMSF) graphs of the *Ld*CRK12–ligand complexes generated over a 10 ns molecular dynamics simulation. (**a**) RMSD versus time graph of *Ld*CRK12–ligand complexes; (**b**) Radius of gyration (Rg) versus time graph of *Ld*CRK12–ligand complexes; and (**c**) Analysis of RMSF trajectories of residues of *Ld*CRK12–ligand complexes. For the 3 graphs, the unbound protein (*Ld*CRK12), compound 5, GSK3186899 (compound 7), compound 8, NANPDB1406, NANPDB1649, NANPDB2581, NANPDB6446, and ZINC000095485940 are represented as black, red, green, blue, yellow, brown, grey, purple and cyan, respectively.

Molecular dynamics studies the motion of atoms along the course of time by the integration of Newton’s equations of motions [101]. Molecular dynamics simulations were performed using GROMACS 2018 to elucidate the dynamic behavior of selected compounds within the active sites of the *Ld*CRK12 protein. The root mean square deviation (RMSD), the radius of gyration (Rg), and root mean square fluctuation (RMSF) were analyzed for the unbound protein and the protein–ligand complexes (Figure 7a–c).

#### 3.11.1. The Root Mean Square Deviation (RMSD) of the Complexes

To evaluate the stability of the *Ld*CRK12–ligand complexes, the RMSD plots of the unbound protein and the *Ld*CRK12–ligand complexes were analyzed (Figure 7a). The RMSD is a frequently used measure of the differences between the structures sampled during the simulation and the reference structure [102]. MD simulations require systems to be close to their native conformation. The time trajectory of RMSD shows the deviation of a protein structure from a reference structure as a function of time [102].

The RMSD values of all nine structures experienced a gradual rise from 0 to 3 ns. The unbound *Ld*CRK12 was observed to rise steadily until about 4 ns and maintained stability until about 5 ns, with an average RMSD of 1.1 nm. The RMSD of the unbound protein rose to an average of 1.25 nm until about 8 ns, and experienced a fall to an average of 1.0 nm until the end of the 10 ns simulation period. The RMSD plot of the *Ld*CRK12-GSK3186899 complex showed a similar trend to that of the unbound *Ld*CRK12. However, the *Ld*CRK12-GSK3186899 complex did not experience a decline at 8 ns, but maintained the average 1.25 nm value until the end of the period. *Ld*CRK12-compound 8 complex demonstrated the highest RMSD values with the most fluctuations. *Ld*CRK12-compound 8 complex demonstrated a steep rise from 0 to 1.6 ns, maintained an average RMSD value of 1.25 nm for about 1.5 ns, and experienced some fluctuations until the end of the 10 ns period (Figure 7a). The *Ld*CRK12-compound 5 complex experienced stability until about 6 ns with an average RMSD of 1.0 ns and rose gradually to an average of 1.25 from 7 ns until the end (Figure 7a).

*Ld*CRK12-NANPDB1406 complex exhibited the lowest RMSD average of 0.8 nm until about 4.2 ns where it rose to 1.2 nm (Figure 7a). The *Ld*CRK12-NANPDB2581 complex experienced the longest stability with an average RMSD value of 0.9 nm until about 7 ns where a gradual rise was observed (Figure 7a). *Ld*CRK12-ZINC000095485940, *Ld*CRK12-NANPDB1649, and *Ld*CRK12-NANPDB6446 complexes were unstable from 0 to about 6 ns where they maintained stable RMSDs with averages of 1.5, 1.55, and 1.5 nm, respectively, until the end of the 10 ns simulation period (Figure 7a).

#### 3.11.2. The Radius of Gyration (Rg) of Complexes

This study analyzed the compactness and folding of the unbound protein and the protein–ligand complexes by plotting the radius of gyration over simulation time. The loss of compactness affects the stability of the complex by introducing weak intermolecular bonds. When the Rg of a complex is higher, the compactness of the protein–ligand complex is lower, causing the interactions between ligand and protein to be weaker [103]. A stably folded protein will maintain a relatively steady Rg while the Rg value is likely to change over time if the protein unfolds [104].

The Rg values of the unbound *Ld*CRK12 and all the eight *Ld*CRK12–ligand complexes ranged between 3.9 nm and 4.9 nm (Figure 7b). The Rg of the *Ld*CRK12 experienced a decline from 0 ns to about 5.5 ns, maintained a steady Rg of an average of 4.05 nm until about 8.6 ns, and experienced a rise to 4.2 nm (Figure 7b). *Ld*CRK12-compound 5 and *Ld*CRK12-GSK3186899 complexes exhibited similar Rg trends as that of the unbound *Ld*CRK12. Both complexes also exhibited relatively lower Rg values than that of the unbound protein throughout the 10 ns simulation period. Both complexes experienced a fall in Rg values until about 4 ns. The Rg of *Ld*CRK12-compound 5 complex was observed to fall to about 5.8 ns and maintained a steady Rg average of 4 nm till the end of the 10 ns period. The Rg of the *Ld*CRK12-GSK3186899 complex rose at 5 ns and maintained an average Rg value of 4.1 nm until the end of the simulation period (Figure 7b).

The Rg values of the *Ld*CRK12-compound 8, *Ld*CRK12-NANPDB6446, *Ld*CRK12-ZINC000095485940, *Ld*CRK12-NANPDB2581 and *Ld*CRK12-NANPDB1649 complexes experienced various degrees of fluctuations due to unstable Rg values (Figure 7b). The *Ld*CRK12-NANPDB1406 complex demonstrated the most stable Rg from 0 to 8 ns with an average of 4.2 ns, which then rose to 4.35 ns for about 1.5 ns and experienced a sharp rise to 4.6 nm.

#### 3.11.3. The Root Mean Square Fluctuation (RMSF) of the Complexes

The RMSF trajectories of the unbound *Ld*CRK12 structure and *Ld*CRK12–ligand complexes were also investigated. The RMSF reveals the flexibility of different regions of a protein, which can be related to crystallographic B-factors [102]. Residues contributing to the complex structural fluctuation can be assessed by this stability profile analysis. Higher RMSF values imply greater fluctuations. Protein regions involved in ligand binding and catalysis are known to demonstrate greater fluctuations [105]. Adaptive variation in flexibility lies principally in these regions of the protein sequence that affect the conformational stabilities of the protein–ligand complex [105].

The RMSF plots revealed that all eight compounds caused some degree of fluctuations in similar regions of the *Ld*CRK12 (Figure 7c). Fluctuations were observed at regions from residue index 30–50, 60–150, 280–350, and 700–800. The highest fluctuation was observed between residues 60–150 followed by residues 280–350, implying they could be involved in ligand binding.

### 3.12. MM/PBSA Computations

#### 3.12.1. Contributing Energy Terms

The molecular mechanics Poisson–Boltzmann surface area (MM/PBSA) computation was employed to determine the binding free energies of the *Ld*CRK12–ligand complexes. At a quantitative level, simulation-based methods provide substantially more accurate estimates of ligand binding free energies than other computational approaches such as docking [106]. The calculation of the binding free energy ΔG_bind_, which is the free energy difference between the ligand-bound state and the corresponding unbound states of protein and ligand, is used to quantify the affinity of a ligand to its target. Assessing the ΔG_bind_ of a series of ligands against a particular target can reveal those ligands with higher binding affinities to the target. Thus, the ΔG_bind_ calculations are important to gain in-depth knowledge about the binding modes of the hits in drug design [107].

The MM/PBSA calculations showed that compound 8 had the lowest binding free energy of −68.609 kJ/mol (Table 11). Compound 5 was also observed to have a binding free energy of −54.023 kJ/mol while GSK3186899 had −27.382 kJ/mol (Table 11). Compounds 5 and 8 demonstrated better inhibitory activities against *L. donovani* than GSK3186899, although GSK3186899 was selected as the preclinical candidate due to pharmacokinetics and safety concerns [20]. NANPDB1649 had the lowest binding free energy of −50.434 kJ/mol among the five selected hits (Table 11). NANPDB2581, NANPDB6446 and NANPDB1406 also demonstrated low binding free energies of −49.374, −37.179 and −24.518 kJ/mol, respectively. These compounds exhibited binding affinities similar or better than that of the preclinical candidate (GSK3186899), thus are worthy of further experimental validation.

Even though ZINC000095485940 was predicted to have the lowest binding energy to the *Ld*CRK12 (−10.1 kcal/mol) by Autodock Vina, it had the highest binding free energy of 0.593 kJ/mol from the MM/PBSA computations (Table 11), thereby potentially limiting its lead-likeness. In a previous study, compounds with high binding free energies have been shown to demonstrate inhibitory activity against receptors due to their very low electrostatic energies and very high polar energies [108]. ZINC000095485940 demonstrated high polar solvation energy of 136.331 kJ/mol and electrostatic energy of −29.485 kJ/mol (Table 11).

Previous studies have reported that electrostatic and van der Waals forces contribute predominantly and continuously to the binding energy along with simulations that favored the binding of complexes [26,109]. All compounds demonstrated very low van der Waal’s energies, ranging from −84.419 kJ/mol to −138.191 kJ/mol (Table 11).

#### 3.12.2. Per-Residue Energy Decomposition

**Figure 8:**
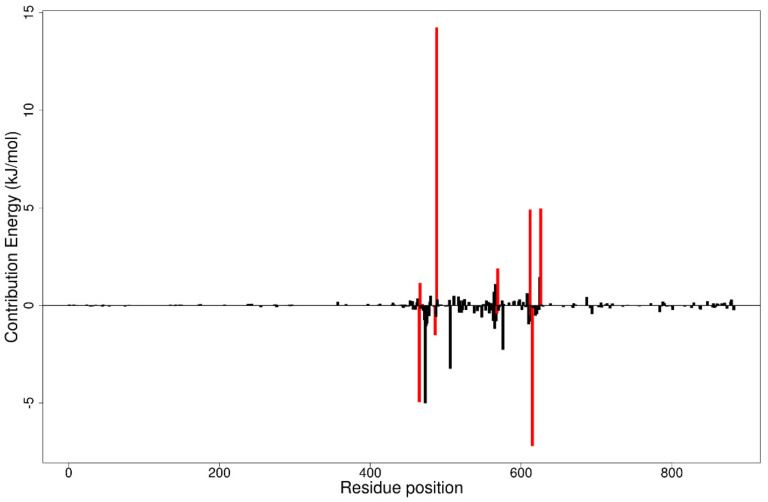
Molecular mechanics Poisson–Boltzmann surface area (MM/PBSA) plot of binding free energy contribution per residue of the *Ld*CRK12-NANPDB1406 complex.

The MM/PBSA method can be used to calculate free binding energies by per-residue decomposition. This involves the decomposition of each residue by including the interactions in which each residue is involved. These provide useful insight into important interactions of key residues in free energy contribution. Residues contributing binding free energy greater than 5 kJ/mol or less than −5 kJ/mol are worth considering as key residues for the binding of a ligand to a protein [110]. The per-residue energy decomposition computations for each complex were performed (Figure 8 and Figure S4A–G).

From the protein–ligand interactions, residues Leu465, Ser466, Thr469, Ala486, Lys488, Ala568, Ser569, Asp612, Asn613, Leu615, and Asp626 were considered as key residues for ligand binding in the ATP binding site (Section 3.6). From the MM/PBSA per residue decomposition computations for the *Ld*CRK12-GSK3186899 complex, it was observed that only Lys488 and Arg575 contributed individual energies beyond the ±5 kJ/mol threshold with energy values of 10.1287 and 5.8145 kJ/mol, respectively (Figure S4B). For the *Ld*CRK12-NANPDB1406 complex, Val473, Lys488 and Leu615 contributed energies of −5.0135, 14.2430, and −7.2060 kJ/mol, respectively (Figure 8). Only Lys488 was observed to contribute individual energy above the ±5 kJ/mol threshold with values of 7.8042 and 13.3733 kJ/mol in the *Ld*CRK12-NANPDB1649 and *Ld*CRK12-NANPDB2581 complexes, respectively (Figure S4D,E). Additionally, Asp612 was the only residue that contributed individual energy beyond the ±5 kJ/mol with an energy value of 5.4536 kJ/mol in the *Ld*CRK12-NANPDB6446 complex (Figure S4F). For the *Ld*CRK12-ZINC000095485940 complex, Lys488 and Asp626 contributed 17.8578 and 9.9136 kJ/mol, respectively (Figure S4G). From the per-residue energy decomposition computations, it is suggested that Lys488 is a very crucial residue for ligand binding in the ATP binding site, which warrants further experimental validation to determine its role.

For the ligand binding in pocket 14, residues Leu723, Gly724, Pro725, Leu726, Pro727, Pro728, Val731, Leu743, Asn763, Trp764, and Gln815 were identified as key. From the MM/PBSA per residue decomposition of *Ld*CRK12-compound 8, Pro728 and Trp764 were observed to contribute energies beyond the ±5 kJ/mol threshold with individual energy values of −6.7576 and −6.5709 kJ/mol, respectively (Figure S4C). No residue was observed to contribute energy beyond the ±5 kJ/mol threshold in the *Ld*CRK12-compound 5 complex (Figure S4A).

### 3.13. Future Outlook and Implication of the Study

This study modelled a reasonable structure of *Ld*CRK12 with good quality parameters which has been made available to the scientific community to enrich work on structure-based drug discovery. Additionally, small molecules with the potential to inhibit the activity of *Ld*CRK12 were identified, which could serve as the building blocks for the design of novel biotherapeutics. The study further proposed suitable molecules with negligible toxicity. Since the study is entirely computational, making available structures and compounds enable synthesis and screening to ascertain their potency as antileishmanial molecules. These predicted compounds can help stimulate the pace of searching for effective antileishmanial drugs globally.

In order to identify polypharmacological agents against leishmaniasis, it warrants investigating the inhibitory potential of the identified biomolecules against other CDC-2-related kinases of *Leishmania*, especially CRK3 and CRK6 [111]. CRK3 is essential for cell cycle progression and growth in *Leishmania mexicana* [112,113], while the role of CRK6 remains unclear [113,114], it has accessory functions in the cell cycle in *T. brucei* [114].

## 4. Conclusions

Natural products have shown the potential to be repurposed as effective *L. donovani* CRK12 inhibitors. This study sought to identify potential *Leishmania* inhibitors from the African flora by targeting the *Ld*CRK12. The study identified four potential bioactive compounds comprising NANPDB1406, NANPDB2581, NANPDB6446 and NANPDB1649 with binding affinities of −9.5, −9.2, −9.1 and −8.5 kcal/mol, respectively. NANPDB1406, NANPDB2581 and NANPDB6446 demonstrated higher binding affinities than the preclinical compound (GSK3186899) which had the binding energy of −8.5 kcal/mol [20]. This study suggests Lys488 as a very crucial residue for ligand binding in the ATP binding site. MD simulations, including MM/PBSA, corroborated the potential inhibition of *Ld*CRK12 by the compounds. Physiochemical and toxicological profiling predicted the compounds to be drug-like and have insignificant toxicity concerns. Ligand quality metrics comprising inhibitory constant (Ki), ligand efficiency (LE), fit quality (FQ), LE scale (LE_scale), and LE-dependent lipophilicity (LELP) also indicated that the potential antileishmanial compounds could serve as templates for fragment-based drug design for *Leishmania* inhibitors. The predicted Ki values of the potential drug candidates ranged from 0.108 to 0.587 μM. Furthermore, the molecules were predicted as antileishmanial molecules, necessitating experimental evaluation to corroborate their bioactivity.

## Figures and Tables

**Table 1:** BLAST results showing identical proteins to the *Ld*CRK12. The best template is selected based on the E-value, sequence identity, BLAST score, and availability of a 3D structure.

ID	Protein Name	E-Value	BLAST Score	Identity (%)
O14098	C-terminal heptapeptide repeat domain CTD kinase subunit alpha (Schizosaccharomyces pombe (strain 972/ATCC 24843) (Fission yeast))	4.2 × 10^−34^	356	36
Q9TVL3-2	Isoform a, of Probable cyclin-dependent kinase 9 (Caenorhabditis elegans)	9.7 × 10^−34^	348	35
Q641Z4	Cyclin-dependent kinase 9 (Rattus norvegicus)	7.4 × 10^−34^	345	31.3
Q99J95	Cyclin-dependent kinase 9 (Mus musculus)	7.4 × 10^−34^	345	31.3
P50750	Cyclin-dependent kinase 9 (Homo sapiens)	7.4 × 10^−34^	345	31.3
Q5EAB2	Cyclin-dependent kinase 9 (Bos taurus)	1.4 × 10^−33^	343	31

**Table 2:** Discrete optimized protein energy (DOPE) and GA341 scores of the 5 generated models using Modeller 9.2.

Models	Dope Score	GA341 Score
MOD1	−49,545.96484	0.36807
MOD2	−49,137.34766	0.20576
MOD3	−47,466.54688	0.10907
MOD4	−49,459.57422	0.28138
MOD5	−50,486.88281	0.21007

**Table 3:** Predicted I-TASSER models and C-scores.

Models	ITAS1	ITAS2	ITAS3	ITAS4	ITAS5
C-Score	−3.68	−3.85	−2.87	−2.77	−2.66

**Table 4:** Model evaluation of the Robetta predicted models using SAVES v5.0.

Models	Model Score			
	Verify (%)	Errat	Prove (%)	Procheck
ROB1	82.97	88.0579	0.0 (Pass)	4E, 3W and 2P
ROB2	66.06	87.4259	0.0 (Pass)	5E, 2W and 2P
ROB3	65.83	84.7073	6.1 (Error)	5E, 1W and 3P
ROB4	67.54	83.6538	6.8 (Error)	5E, 2W and 2P
ROB5	78.55	87.822	5.8 (Error)	5E, 0W and 4P

**Table 5:** Model evaluation of the top 3 *Ld*CRK12 structures modelled via Modeller, Robetta, and I-TASSER. Models ROB1, ITAS5, and MOD5 were generated using Robetta, I-TASSER, and Modeller, respectively. E: error; W: warning; and P: pass.

Tool	Model Score		
	ROB1	ITAS5	MOD5
Verify (%)	82.97	85.36	41.20
Errat (Quality Factor)	88.0579	80.2158	10.0536
Prove (%)	0.0 (Pass)	9.5 (Error)	16.1 (Error)
Procheck	4E, 3W and 2P	6E, 2W and 1P	5E, 2W and 1P

**Table 6:** Ramachandran plot statistics for the best models from the 3 modelling techniques. For all 3 models, the number of end residues (excluding Gly and Pro) = 2, Glycine residues = 65, Proline residues = 85, and the total number of residues = 881.

Model	MOD5		ITAS5		ROB1	
	No. of Residues	Percentage	No. of Residues	Percentage	No. of Residues	Percentage
Most favored regions [A, B, L]	581	79.7	445	61.0	598	82.0
Additionally allowed regions [a, b, l, p]	113	15.5	217	29.8	125	17.1
Generously allowed regions [~a, ~b, ~l, ~p]	22	3.0	43	5.9	3	0.4
Disallowed regions	13	1.8	24	3.3	3	0.4
Non-glycine and non-proline residues	729	100.0	729	100.0	729	100.0

**Table 7:** Predicted binding sites located around the kinase domain of the *Ld*CRK12. Solvent accessible (SA) values are shown.

Pocket	Area (SA)/Å2	Volume (SA)/Å3	Residues Lining the Pocket
1	566.585	712.561	Leu438, Pro439, Ala441, Pro442, Pro443, Pro444, Ser445, Glu463, Lys464, Leu465, Ser466, Glu467, Gly468, Thr469, Tyr470, Val473, Lys475, Ala486, Leu487, Lys488, Glu506, Leu510, Ser544, Phe563, Ala564, Tyr565, Ala566, Thr567, Ala568, Ser569, Ala571, Gly572, Arg575, Arg576, His606, Asp608, Lys610, Asp612, Asn613, Leu615, Thr625, Asp626, Phe627, Leu629, Cys630, Val650, Thr652
2	312.963	420.314	Met492, Thr495, His496, Gly498, Phe499, Pro500, Gln501, Thr502, Arg505, Arg607, Gly628, Leu629, Cys630, Ser631, Arg639, Cys640, Val644, Thr647, Pro648, Ser649, Val650, Ile651, Arg656, Met660, Thr665, Tyr667, Ser708, Ala709, Glu712
3	443.095	377.107	Ile536, Arg597, Lys598, His600, Glu601, Arg603, Pro635, Asp668, Glu669, Lys670, Thr823, Ala825, Glu826, Leu828, Arg829, Leu836, Asp837, Asp838, Ala839, Pro840, Leu841, Leu842, Tyr845, Gln846, Arg847, Val848, Leu849
4	207.834	203.064	Arg692, His693, Ala695, Gln696, Gln699, Gln700, Arg703, Pro705, Thr711, Glu714, Gln715, Ser717, Thr720, Glu721, Gln749, Ala758, Ala759, Gln760, Ala762
5	110.211	105.944	Thr720, Pro725, Leu726, Pro727, Pro728, Val731, Leu743, Leu746, Glu747, Gln749, Gly750, Arg751, Glu754, Pro761, Ala762, Asn763
6	141.403	95.351	Ala571, Arg575, Lys610, Ser611, Asp612, Thr652, Ala654, Tyr655, Gln682, Leu686, Glu687, Pro688, Tyr691, Arg694, Phe780
7	50.346	89.822	Pro635, Gly636, Ser637, Leu849, Pro850, Thr852
8	159.056	87.650	Leu685, Glu687, Pro688, Pro689, Tyr691, Arg692, Arg694, Ala695, Gln698, Gln699, Arg718, Glu721, Ser774, Phe775, Leu776, Gln778, Gln779, Phe780
9	101.847	84.378	Ala342, Val402, Ala403, Met404, Gly405, Leu412, Arg413, Leu415, Pro417, Tyr420, Arg429
10	60.970	41.895	Phe580, Glu584, Leu587, Leu588, Lys591, Glu619, Gly620, Val622
11	150.732	38.172	Cys574, Phe578, Ala579, Phe580, Thr581, Pro582, Met585, Gln682, Met683, Phe684, Leu686, Ile770, Phe771, Gly785, Trp786, Glu788, Glu790, His799, Arg801, Pro802
12	68.900	28.853	Thr642, His643, Val644, Pro658, Glu659, Leu662, Gly663, Ser664, Leu726, Ser736, His739, Met740, Leu816, Pro818, Arg821
13	69.295	22.359	Pro427, Arg429, Arg430, Val434, Gly435, Phe448, Gln452, Lys456
14	71.553	21.095	Pro658, Leu662, Leu713, Leu716, Ser717, Ile719, Thr720, Gly724, Pro725, Leu726, Val742, Gln745, Leu746, Gln749, Leu816

**Table 8:** The binding energies and intermolecular bonds between *Ld*CRK12 and selected compounds.

Compound	Binding Energy (kcal/mol)		Hydrogen Bonds (Bond Length (Å))		Hydrophobic Bonds	
	*Ld*CRK12	CDK9	*Ld*CRK12	CDK9	*Ld*CRK12	CDK9
ZINC000095485940	−10.1	−7.7	Gly468 (2.93), Ser569 (2.95), Asp626 (2.70)	Arg195 (3.06, 3.22), Glu234 (2.9), Arg343 (3.1)	Leu465, Ser466, Thr469, Val473, Ala486, Lys488, Ser544, Phe563, Asp612, Asn613, Leu615, Thr625	Arg188, Leu192, Arg195, Thr233, Glu234, Tyr338, Ala340, Arg343
NANPDB1406	−9.5	−7.3	Lys488 (3.26), Ala566 (2.89, 2.97, 3.07), Ser569 (3.01)	Asn232 (2.8), Phe336 (3.08), Ala340 (2.69, 3.15), Arg343 (2.88, 3.1, 3.17, 3.25)	Leu465, Ser466, Gly468, Val473, Ala486, Tyr565, Thr567, Ala568, Asp612, Leu615, Asp626	Asn232, Thr233, Met335, Phe336, Tyr338, Ala340, Arg343
NANPDB2581	−9.2	−7.5	Lys610 (3.08)	Arg195 (3.32), Arg343 (3.09, 3.35)	Leu465, Ser466, Thr469, Tyr470, Ala568, Ser569, Asp612, Asn613, Leu615, Asp626	Leu192, Arg195, Thr233, Glu234, Pro341, Pro342, Arg343
NANPDB6446	−9.1	−7.3	Ser569 (2.77, 3.02), Arg575 (2.87, 3.15)	Asn179 (3.04), Tyr259 (2.89)	Leu465, Ser466, Ala568, Gly572, Asp612, Asp626	Asn179, Pro182, Glu203, Asp205, Trp253, Asn258, Tyr259, Pro300
Compound 8	−9.1	−9.0	Leu723 (2.83)	Lys48 (2.91), Asp149 (2.99, 3.08)	Gly724, Pro725, Leu726, Pro727, Pro728, Val731, Leu743, Glu747, Asn763, Trp764, Gln815, Leu816	Thr29, Phe30, Leu51, Pro60, Thr62, Asp149, Leu170, Arg188, Val190, Thr191, Leu192, Met335
T6Q	−9.1	−8.6	Thr469 (3.06)	-	Leu465, Ser466, Gly468, Thr469, Val473, Lys488, Ala568, Ser569, Arg575, Lys610, Asp612, Asn613, Leu615, Asp626	Ile25, Phe30, Val33, Ly48, Asp109, Gly112, Ala153, Leu156, Ala166, Asp167, His331, Leu332, Thr333
DDD853651/GSK3186899/Compound 7	−8.5	−8.8	Ser466 (2.96), Gly468 (3.19), Lys488 (3.03), Ser544 (3.27), Thr625 (3.12), Asp626 (3.31, 3.3), Tyr691 (2.98)	Glu107 (3.07, 2.98)	Gly468, Thr469, Tyr470, Val473, Ala486, Lys488, Phe563, Lys610, Asp612, Leu615, Asp626, Tyr691	Ile25, Val33, Lys35, Lys48, Phe103, Glu107, His108, Asp109, Ala166, Asp167
Compound 5	−7.2	−8.6	Leu723 (2.98, 3.07)	Cys106 (3.2, 3.0)	Gly724, Pro725, Leu726, Pro727, Pro728, Val731, Tyr732, Leu743, Asn763, Trp764, Gln815, Leu816, Asp817, Gln820	Ile25, Val33, Ala46, Lys48, Phe103, Phe105, Glu107, His108, Asp109, Gly112, Leu113, Ala153, Asn154, Leu156, Ala166, Asp167

**Table 9:** Ligand quality assessment metrics for selected compounds. The metrics include inhibitory constant (Ki), ligand efficiency (LE), LE scale (LE_scale), fit quality (FQ), LE-dependent lipophilicity (LELP), and calculated logP (cLogP).

Compound	Binding Energy	NHA	cLogP	Ki (µM)	LE	LE_Scale	FQ	LELP
ZINC000095485940	−10.1	29	−0.1814	0.039	0.348	0.347	1.003	0.521
NANPDB1406	−9.5	23	1.5531	0.108	0.413	0.416	0.993	3.761
NANPDB2581	−9.2	24	3.3633	0.180	0.383	0.404	0.948	8.781
NANPDB6446	−9.1	26	−0.8296	0.213	0.35	0.380	0.921	2.370
NANPDB1649	−8.5	26	3.2246	0.587	0.327	0.380	0.861	9.861

**Table 10:** Selected compounds and known *Ld*CRK12 inhibitors with their two-dimensional (2D) structures and common names or International Union of Pure and Applied Chemistry (IUPAC) names. The IUPAC names were generated using the Marvin suite (http://www.chemaxon.com/; accessed on 27 February 2020).

Compound ID	Common/IUPAC Name	2D Structure
ZINC000095485940	(1R,2R,4R,7S,8R,10R,11R,12R,13R,16S)-7-(furan-3-yl)-10,13-dihydroxy-8,13-dimethyl-3,6,14-trioxapentacyclo[9.7.0.02,4.02,8.012,16]octadecane-5,18-dione	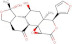
NANPDB1406	methyl ellagic acid	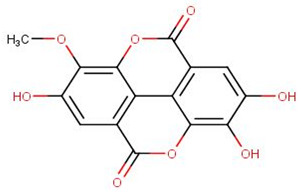
NANPDB2581	stylopine	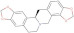
NANPDB6446	sennecicannabine	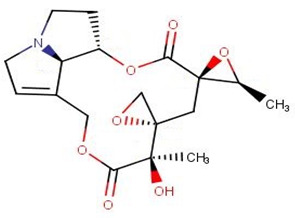
NANPDB1649	sesamin	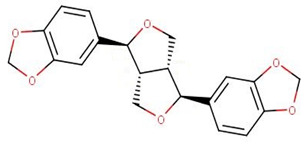
Compound 5	2-methyl-N-[(1r,4r)-4-{[3-(2-methoxyphenyl)-1H-pyrazolo[3,4-d]pyrimidin-6-yl]amino}cyclohexyl]propane-1-sulfonamide	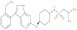
GSK3186899	DDD853651/Compound 7	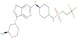
Compound 8	2-(2,4-difluorophenyl)-1-(4-{[3-(2-methoxyphenyl)-1H-pyrazolo[3,4-d]pyrimidin-6-yl]amino}piperidin-1-yl)ethan-1-one	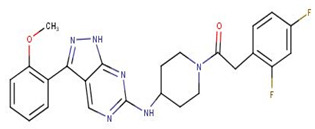

**Table 11:** Contributing energy terms of the MM/PBSA computations for the *Ld*CRK12–ligand complexes. Energy values are shown as average ± standard deviations in kJ/mol. SASA denote solvent accessible surface area.

	van der Waal Energy (kJ/mol)	Electrostatic Energy (kJ/mol)	Polar Solvation Energy (kJ/mol)	SASA Energy (kJ/mol)	Binding Energy (kJ/mol)
Compound 5	−98.909 ± 22.552	−9.113 ± 13.340	67.471 ± 24.307	−13.471 ± 2.359	−54.023 ± 17.067
DDD853651/GSK3186899/Compound 7	−107.423 ± 24.517	−43.202 ± 23.069	140.188 ± 36.187	−16.945 ± 2.444	−27.382 ± 20.792
Compound 8	−138.191 ± 15.201	−17.732 ± 9.037	103.997 ± 24.258	−16.683 ± 1.392	−68.609 ± 13.327
NANPDB1406	−125.840 ± 9.460	−40.995 ± 10.072	157.502 ± 22.889	−15.186 ± 0.971	−24.518 ± 14.412
NANPDB1649	−111.638 ± 18.534	−4.178 ± 8.634	80.033 ± 25.137	−14.651 ± 1.960	−50.434 ± 13.538
NANPDB2581	−110.229 ± 10.366	−7.999 ± 7.595	83.680 ± 20.147	−14.826 ± 1.181	−49.374 ± 14.169
NANPDB6446	−84.419 ± 19.455	−64.626 ± 32.749	125.008 ± 32.850	−13.141 ± 2.305	−37.179 ± 17.980
ZINC000095485940	−91.882 ± 13.394	−29.485 ± 12.960	136.331 ± 31.350	−14.372 ± 1.704	0.593 ± 16.180

## Data Availability

Not applicable.

## Supplementary Materials

The following are available online at https://www.mdpi.com/2218-273X/11/3/458/s1, Figure S1: ERRAT error plots of the selected models: (A) ERRAT error plot for MOD5, (B) ITAS5, and (C) ROB1. Red bars represent the misfolded regions, yellow bars demonstrate the error region between 95% and 99%, and green bars indicate the region with a lower error rate for protein folding, Figure S2: Ramachandran plots of the protein structures obtained via PROCHECK. (A) Ramachandran plot of protein model MOD5 (B) Ramachandran plot of protein model ITAS5. The percentages of residues of model MOD5 in the most favored regions, additionally allowed regions, generously allowed regions and disallowed regions are 79.7, 15.5, 3.0 and 1.8%, respectively. For model ITAS5, 61.0, 29.8, 5.9 and 3.3% of the amino acid residues were predicted to be in the most favored, additionally allowed, generously allowed and disallowed regions, respectively, Figure S3: The 2D diagrams of the *Ld*CRK12–ligand interaction generated using LigPlot+. Interaction profiles of (A) *Ld*CRK12-ZINC000095485940 complex; (B) *Ld*CRK12-NANPDB1406 complex; (C) *Ld*CRK12-NANPDB2581 complex and (D) *Ld*CRK12-GSK3186899 complex. The ligands are colored in purple, hydrogen bonds are represented as green dash lines and hydrophobic contacts are represented as red spoke arcs, Figure S4: Molecular mechanics Poisson–Boltzmann surface area (MM/PBSA) plot of binding free energy contribution per residue of protein–ligand complexes (A) *Ld*CRK12-compound 5 (B) *Ld*CRK12-compound 7; (C) *Ld*CRK12-compound 8; (D) *Ld*CRK12-NANPDB1649 (E) *Ld*CRK12-NANPDB2581 (F) *Ld*CRK12-NANPDB6446 (G) *Ld*CRK12-ZINC000095485940, Table S1: The binding energies and intermolecular bonds between *Ld*CRK12 and compounds, Table S2: ADME Prediction of top 19 hits and known drugs for Gastrointestinal (GI); Blood Brain Barrier (BBB); Estimated Solubility (ESOL) class, P-glycoprotein (Pgp) and TPSA, Table S3: Toxicological profiles of the 17 hits and the known drugs, Table S4: Predicted biology activity of the lead compounds using Prediction of Activity Spectra for Substances (PASS). Pa and Pi represent probable activity and probable inactivity, respectively. Supplementary_file_1: 3D model of the *Ld*CRK12 generated using Modeller (MOD5). Supplementary_file_2: The 3D model of the *Ld*CRK12 generated using I-TASSER (ITAS5). Supplementary_file_3: The 3D model of the *Ld*CRK12 generated using Robetta (ROB1).
