# Supplementary material for: A Molecular Modeling Approach to Identify Potential Antileishmanial Compounds Against the Cell Division Cycle (cdc)-2-Related Kinase 12 (CRK12) Receptor of Leishmania donovani
Source: Biomolecules. 2021 Mar 18;11(3):458. doi: 10.3390/biom11030458 (PMC8003136; doi:10.3390/biom11030458)
Supplement: Supplementary file 1 [file biomolecules-11-00458-s001.zip › Supplementary_EB.pdf]

Table S1: The binding energies and intermolecular bonds between *Ld*CRK12 and compounds.

Compound	Binding Energy (kcal/mol)		HYDROGEN BONDS [BOND LENGTH (Å)]		HYDROPHOBIC BONDS	
	***Ld*CRK12**	**CDK9**	**LdCRK12**	**CDK9**	***Ld*CRK12**	**CDK9**
ZINC000095485940	-10.1	-7.7	Gly468 (2.93), Ser569 (2.95), Asp626 (2.70)	Arg195 (3.06, 3.22), Glu234 (2.9), Arg343 (3.1)	Leu465, Ser466, Thr469, Val473, Ala486, Lys488, Ser544, Phe563, Asp612, Asn613, Leu615, Thr625	Arg188, Leu192, Arg195, Thr233, Glu234, Tyr338, Ala340, Arg343
NANPDB1406	-9.5	-7.3	Lys488 (3.26), Ala566 (2.89, 2.97, 3.07), Ser569 (3.01)	Asn232 (2.8), Phe336 (3.08), Ala340 (2.69, 3.15), Arg343 (2.88, 3.1, 3.17, 3.25)	Leu465, Ser466, Gly468, Val473, Ala486, Tyr565, Thr567, Ala568, Asp612, Leu615, Asp626	Asn232, Thr233, Met335, Phe336, Tyr338, Ala340, Arg343
NANPDB2581	-9.2	-7.5	Lys610 (3.08)	Arg195 (3.32), Arg343 (3.09, 3.35)	Leu465, Ser466, Thr469, Tyr470, Ala568, Ser569, Asp612, Asn613, Leu615, Asp626	Leu192, Arg195, Thr233, Glu234, Pro341, Pro342, Arg343
NANPDB6446	-9.1	-7.3	Ser569 (2.77, 3.02), Arg575 (2.87, 3.15)	Asn179 (3.04), Tyr259 (2.89)	Leu465, Ser466, Ala568, Gly572, Asp612, Asp626	Asn179, Pro182, Glu203, Asp205, Trp253, Asn258, Tyr259, Pro300
Compound 8	-9.1	-9.0	Leu723 (2.83)	Lys48 (2.91), Asp149 (2.99, 3.08)	Gly724, Pro725, Leu726, Pro727, Pro728, Val731, Leu743, Glu747, Asn763, Trp764, Gln815, Leu816	Thr29, Phe30, Leu51, Pro60, Thr62, Asp149, Leu170, Arg188, Val190, Thr191, Leu192, Met335
T6Q	-9.1	-9.2	Thr469 (3.06)	Glu203 (2.99), Asn255 (3.07)	Leu465, Ser466, Gly468, Thr469, Val473, Lys488, Ala568, Ser569, Arg575, Lys610, Asp612, Asn613, Leu615, Asp626	Pro182, Glu198, Gly202, Asp205, Tyr206, Gly207, Tyr259, Glu198, Trp253, Tyr259, Glu260, Pro300
T6Q (2nd pose with CDK9)	-	-8.6	-	-	-	Ile25, Phe30, Val33, Ly48, Asp109, Gly112, Ala153, Leu156, Ala166, Asp167, His331, Leu332, Thr333
T6Q (CDK9-T6Q complex as obtained from RCSB PDB)	-	-	-	Cys106 (2.76, 3.02)	-	Ile25, Phe30, Val33, Ala46, Val79, Phe103, Asp104, Phe105, Glu107, Asp109, Ala153, Asn154, Leu156, Asp167
NANPDB267	-9	-7.6	Lys488 (3.04, 3.24),	Arg343 (2.85,	Leu465, Ser466, Gly468,	Asn232, Thr233,

			Asp626 (3.05)	3.05, 3.06, 3.12, 3.2)	Tyr470, Val473, Ala566, Thr567, Ala568, Ser569, Asp612, Leu615	Met335, Phe336, Tyr338, Ala340, Arg343
NANPDB2521	-8.7	-6.8	Gly636 (3.01), Thr642 (2.77), His643 (2.94), Gly663 (3.00), Tyr667 (2.80), Asp668 (2.75, 3.16)	Thr233 (3.02), Tyr338 (3.07), Ala340 (2.91), Arg343 (2.85, 3.2, 3.26)	Pro635, Glu659, Leu662, Ser664, Thr665, Ala666, Met740, Pro818	Thr233, Phe336, Tyr338, Leu339, Ala340, Pro342, Arg343
NANPDB2966	-8.7	-8	Gln819 (3.21)	-	Thr642, His643, Glu659, Ser664, Asp668, Ser730, His733, Pro818	Arg188, Leu192, Thr233, Met335, Tyr338, Leu339, Ala340, Pro342, Arg343
NANPDB3435	-8.7	-8.1	Thr642 (3.02), Thr665 (2.79, 2.89, 3.05), Ala666 (3.1), Tyr667 (3.17), Gln819 (2.97)	Arg195 (3.1), Glu234 (2.83), Ala340 (2.91, 2.96, 3.21)	Glu659, Ser664, Asp668, Ser730, His733, Met740, Pro818	Thr233, Glu234, Met335, Phe336, Tyr338, Leu339, Ala340, Pro342, Arg343
NANPDB3284	-8.6	-6.6	Ala566 (2.8, 3.06), Ser569 (3.01, 3.04)	Asn232 (3.28), Phe336 (2.92), Ala340 (2.93), Arg343 (2.88, 3.06, 3.24)	Leu465, Ser466, Ala486, Lys488, Tyr565, Ala568, Asp612, Leu615	Asn232, Thr233, Met335, Phe336, Tyr338, Ala340, Arg343
NANPDB4609	-8.6	-6.6	Leu465 (2.7), Ala566 (3.15), Thr567 (2.83), Ser569 (2.74, 3.13)	Gly207 (3.23, 3.24), Trp253 (3.15, 3.15), Pro254 (3.17), Tyr259 (3.12)	Ser466, Val473, Ala486, Lys488, Gly572, Ala568, Asp612, Leu615, Thr625, Asp626	Glu198, Glu203, Asp205, Tyr206, Gly207, Pro209, Trp253, Asn255, Tyr259, Pro300
NANPDB2582	-8.6	-7.3	Lys488 (3.13)	Arg343 (3.15)	Leu465, Ser466, Gly468, Thr469, Tyr470, Val473, Ala568, Ser569, Asp612, Asp626	Asn232, Thr233, Glu234, Gln235, Met335, Phe336, Tyr338, Pro341, Pro342, Arg343
NANPDB3239	-8.6	-7.7	Ser466 (3.06), Lys488 (3.09), Ala566 (2.97), Asp626 (2.76)	Gly207 (3.19), Asn255 (2.79, 3.11), Tyr259 (2.82, 3.13)	Leu465, Gly468, Val473, Ala486, Thr567, Ala568, Ser569, Gly572, Arg576, Asp612, Leu615	Pro182, Gly202, Glu203, Arg204, Asp205, Tyr206, Asn255, Asn258, Tyr259, Pro300
Amphotericin B	-8.6	-8.1	Arg603 (2.86, 2.8), Pro635 (3.11, 3.0), Tyr845 (2.74), Gln846 (3.04), Arg847 (3.22)	Ala340 (2.84)	Pro635, Gly636, Thr642, His643, Glu669, Lys670, Thr823, Glu826, Tyr845, Gln846, Arg847, Leu849	Val50, Leu51, Glu53, Glu55, Pro60, Thr62, Gly169, Arg188, Leu192, Thr233, Leu339, Ala340, Pro341, Pro342,
ZINC000095486260	-8.5	-6.4	Ser569 (2.96, 2.96, 3.17, 3.25), Asp612 (2.79, 2.88)	Asn179 (3.13), Trp253 (3.0, 3.29), Asn255 (2.88), Tyr259	Leu465, Ser466, Gly468, Val473, Lys488, Ala566, Ala568, Ala571, Leu615, Asp626	Glu203, Asp205, Trp253, Asn255, Asn258, Tyr259, Asp299, Pro300

				(2.96), Asp299 (2.86)		
NANPDB3614	-8.5	-7.4	Lys488 (2.80)	Trp223 (2.92), Arg284 (3.24, 3.29), Asp285 (2.97)	Leu465, Ser466, Tyr470, Val473, Ala486, Ala568, Ser569, Asp612, Asn613, Leu615, Asp626	Phe121, Thr122, Leu123, Ile126, Trp223, Arg225, Val283, Arg284, Asp285
ZINC000000828203	-8.5	-7.4	Lys488 (2.91)	Trp223 (2.8), Arg284 (3.27), Asp285 (2.93)	Leu465, Ser466, Tyr470, Val473, Ala486, Ala566, Ala568, Ser569, Asp612, Asn613, Leu615, Asp626	Phe121, Thr122, Le123, Ile126, Trp223, Arg225, Val283, Arg284, Asp285
NANPDB1011	-8.5	-7.7	Thr642 (3.24), Ser664 (2.94), Thr665 (2.71, 3.23)	Asp205 (2.89), Gly207 (3.3), Asp299 (3.33)	Ala634, Pro635, Gly636, His643, Glu659, Tyr667, Asp668, Lys670, Val729, Ser730, His733, Pro818, Gln819	Pro182, Glu203, Asp205, Tyr206, Pro209, Asn255, Asn258, Tyr259, Pro300, Ala301
NANPDB1649	-8.5	-7.7	Arg575 (3.29)	Gly207 (3.02)	Leu465, Ser466, Glu467, Gly468, Val473, Lys488, Ala568, Ser569, Asp612, Asn613, Leu615, Asp626	Pro182, Glu203, Asp205, Tyr206, Gly207, Trp253, Asn255, Tyr259, Pro300
NANPDB3949	-8.5	-8.1	Leu746 (3.2)	Asn179 (3.07), Asp205 (2.86), Gly207 (2.91)	Pro725, Leu726, Pro727, Pro728, Val731, Leu743, Glu747, Pro761	Phe174, Ser175, Leu176, Ala177, Asp205, Tyr206, Gly207, Pro209, Pro300
ZINC000095485880	-8.5	-8.2	Thr469 (2.73), Ala564 (2.95)	Arg195 (3.23)	Leu465, Ser466, Glu467, Gly468, Val473, Ala486, Lys488, Ala566, Ser569, Arg575, Asp612, Leu615, Thr625, Asp626	Arg188, Leu192, Arg195, Asn232, Thr233, Glu234, Phe336, Tyr338, Ala340, Pro342, Arg343
DDD853651/ GSK3186899/ Compound 7	-8.5	-8.8	Ser466 (2.96), Gly468 (3.19), Lys488 (3.03), Ser544 (3.27), Thr625 (3.12), Asp626 (3.31, 3.3), Tyr691 (2.98)	Glu107 (3.07, 2.98)	Gly468, Thr469, Tyr470, Val473, Ala486, Lys488, Phe563, Lys610, Asp612, Leu615, Asp626, Tyr691	Ile25, Val33, Lys35, Lys48, Phe103, Glu107, His108, Asp109, Ala166, Asp167
Paromomycin	-7.9	-6.5	Leu465 (2.98), Ser466 (3.14), Thr469 (3.16), Lys488 (2.98), Arg575 (2.81), Ser569 (2.7, 3.02, 3.16), Asp612 (2.9), Asn613 (3.04), Asp626 (2.77, 3.09)	Thr29 (2.95, 2.98), Lys48 (3.27, 3.32), Glu66 (2.77, 3.11), Asp167 (3.12), Arg188 (2.81, 2.92, 3.21, 3.22)	Leu465, Ser466, Glu467, Gly468, Thr469, Tyr470, Val473, Lys488, Ser569, Ala571, Gly572, Lys610, Asp612, Leu615, Asp626	Thr29, Phe30, Lys48, Ala63, Glu66, Asp149, Asp167, Gly169, Leu170, Arg188
Compound 5	-7.2	-8.6	Leu723 (2.98, 3.07)	Cys106 (3.2, 3.0)	Gly724, Pro725, Leu726,	Ile25, Val33, Ala46,

					Pro727, Pro728, Val731, Tyr732, Leu743, Asn763, Trp764, Gln815, Leu816, Asp817, Gln820	Lys48, Phe103, Phe105, Glu107, His108, Asp109, Gly112, Leu113, Ala153, Asn154, Leu156, Ala166, Asp167
Miltefosine	-5.0	-5.6	Gly422 (3.05, 3.1)	-	Leu181, Gly344, Ile345, Thr396, Arg397, Ala399, Pro401, Thr418, Pro419, Tyr420, Pro421, Gly422, Tyr428, Arg432	Ile25, Phe30, Val33, Ala46, Lys48, Phe103, Asp109, Ala111, Gly112, Leu156, Ala166, Asp167, Leu332, Thr333

Table S2: ADME Prediction of top 19 hits and known drugs for Gastrointestinal (GI); Blood Brain Barrier (BBB); Estimated Solubility (ESOL) class, P-glycoprotein (Pgp) and TPSA.

**Compound**	**Molecular Weight**	**cLogP**	**ESOL Class**	**GI Absorption**	**BBB permeant**	**Pgp substrate**	**TPSA**
ZINC000095485940	404.414	-0.1814	Soluble	High	No	Yes	118.73
NANPDB1406	316.221	1.5531	Soluble	High	No	No	130.34
NANPDB2581	323.347	3.3633	Moderately soluble	High	Yes	No	40.16
NANPDB6446	365.381	-0.8296	Very soluble	High	No	Yes	101.13
Compound 8	478.49	3.4878	Moderately soluble	High	No	Yes	96.03
T6Q	462.57	3.6685	Moderately soluble	Low	No	Yes	138.31
NANPDB267	317.340	1.6813	Soluble	High	No	No	68.23
NANPDB2521	290.270	1.5087	Soluble	High	No	Yes	110.38
NANPDB2966	438.518	2.5429	Moderately soluble	High	No	Yes	86.11
NANPDB3435	434.399	3.2155	Moderately soluble	High	No	No	133.52

NANPDB3284	258.228	2.2963	Soluble	High	No	No	90.9
NANPDB4609	394.331	-0.456	Soluble	Low	No	No	151.96
NANPDB2582	383.399	2.7549	Moderately soluble	High	Yes	No	66.46
NANPDB3239	446.450	1.5786	Soluble	Low	No	Yes	145.91
Amphotericin B	924.08	0.323	Moderately soluble	Low	No	Yes	319.61
ZINC000095486260	378.376	0.3706	Soluble	High	No	No	137.43
NANPDB3614	364.352	3.5388	Moderately soluble	High	Yes	Yes	63.22
ZINC000000828203	380.351	3.1931	Moderately soluble	High	No	Yes	83.45
NANPDB1011	492.738	4.7447	Moderately soluble	High	No	Yes	90.15
NANPDB1649	354.357	3.2246	Soluble	High	Yes	No	55.38
NANPDB3949	444.610	4.1395	Moderately soluble	High	No	No	72.83
ZINC000095485880	416.559	4.0458	Moderately soluble	High	Yes	Yes	57.53
DDD853651/ GSK3186899/ Compound 7	491.53	1.9811	Moderately soluble	Low	No	Yes	133.51
Paromomycin	615.63	-9.1987	Highly soluble	Low	No	Yes	347.32
Compound 5	458.58	3.0502	Moderately soluble	Low	No	Yes	130.27
Miltefosine	407.57	0.1224	Moderately soluble	Low	No	Yes	68.4

Table S3: Toxicological profiles of the 17 hits and the known drugs.

**Compound**	**Mutagenic**	**Tumorigenic**	**Reproductive Effect**	**Irritant**
ZINC000095485940	none	none	none	none
NANPDB1406	none	none	none	none
NANPDB2581	none	none	none	none
NANPDB6446	high	high	none	high
Compound 8	none	none	none	none
T6Q	none	none	none	none
NANPDB267	none	none	none	none
NANPDB2521	none	none	none	none
NANPDB2966	none	none	none	none
NANPDB3435	none	none	none	none
NANPDB3284	none	none	high	none
NANPDB2582	none	none	none	none
Amphotericin B	none	none	none	none
ZINC000095486260	none	none	none	high
NANPDB3614	none	high	none	none
ZINC000000828203	none	high	none	none
NANPDB1011	none	none	none	none
NANPDB1649	none	none	none	none
NANPDB3949	none	none	none	none
ZINC000095485880	none	none	none	none
DDD853651/ GSK3186899/	none	low	none	none

Compound 7				
Paromomycin	none	none	none	none
Compound 5	none	none	none	none
Miltefosine	none	none	none	none

Table S4: Predicted biology activity of the lead compounds using Prediction of Activity Spectra for Substances (PASS). Pa and Pi represent probable activity and probable inactivity, respectively.

Compound	Pa	Pi	Activity
ZINC000095485940	0.896	0.005	Antineoplastic
	0.602	0.005	Antiprotozoal
NANPDB1406	0.807	0.005	Kinase inhibitor
	0.681	0.029	Antineoplastic
	0.351	0.071	Antiprotozoal (Leishmania)
NANPDB2581	0.415	0.043	Antiprotozoal (Leishmania)
NANPDB6446	0.793	0.013	Antineoplastic
NANPDB267	0.468	0.013	Antiprotozoal
	0.380	0.112	Antineoplastic
NANPDB2521	0.743	0.009	Kinase inhibitor
	0.398	0.049	Antiprotozoal (Leishmania)
	0.315	0.080	Dermatologic
NANPDB2966	0.932	0.005	Antineoplastic
	0.709	0.004	Antiprotozoal
	0.435	0.037	Antiprotozoal (Leishmania)
NANPDB3435	0.753	0.018	Antineoplastic
	0.700	0.012	Kinase inhibitor
	0.600	0.014	Antiprotozoal (Leishmania)
	0.420	0.049	Dermatologic
NANPDB3284	0.696	0.013	Kinase inhibitor
	0.701	0.026	Antineoplastic
	0.371	0.061	Dermatologic
NANPDB2582	0.366	0.064	Antiprotozoal (Leishmania)
	0.399	0.105	Antineoplastic
ZINC000095486260	0.798	0.012	Antineoplastic
	0.437	0.036	Antiprotozoal (Leishmania)
	0.439	0.044	Dermatologic
	0.344	0.102	Kinase inhibitor
NANPDB3614	0.741	0.020	Antineoplastic
	0.599	0.014	Antiprotozoal (Leishmania)
ZINC000000828203	0.801	0.012	Antineoplastic
	0.363	0.065	Antiprotozoal (Leishmania)
NANPDB1011	0.913	0.005	Antineoplastic

	0.604	0.015	Dermatologic
NANPDB1649	0.787	0.013	Antineoplastic
	0.391	0.052	Antiprotozoal (Leishmania)
NANPDB3949	0.895	0.005	Antineoplastic
	0.696	0.009	Antiprotozoal (Leishmania)
	0.504	0.031	Dermatologic
ZINC000095485880	0.637	0.037	Antineoplastic

(A)

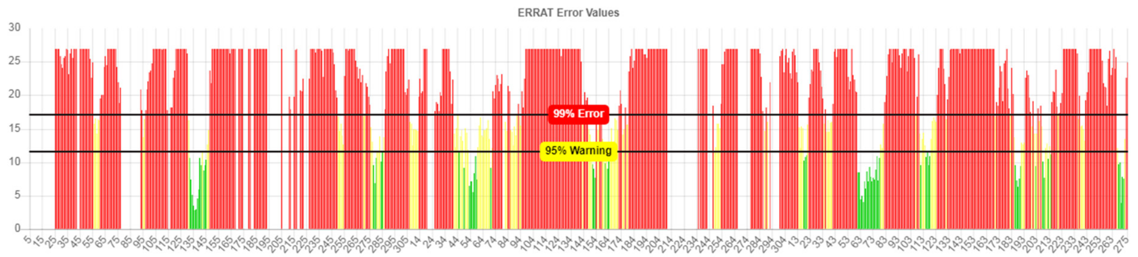

(B)

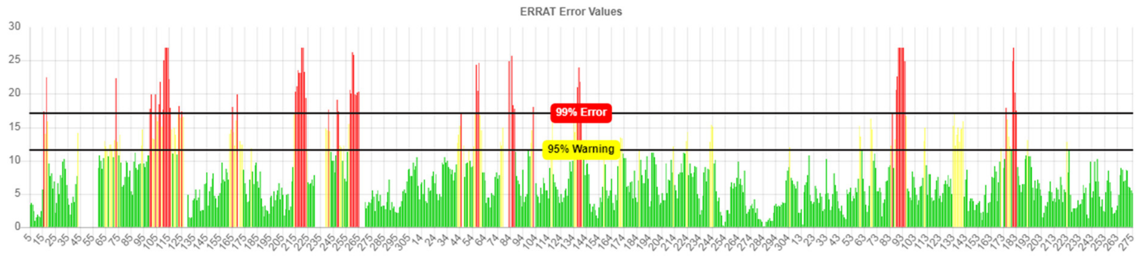

(C)

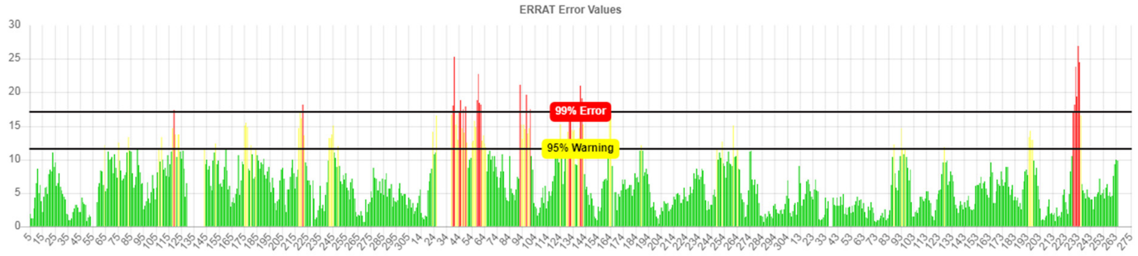

Figure S1: ERRAT error plots of the selected models: (A) ERRAT error plot for MOD5, (B) ITAS5, and (C) ROB1. Red bars represent the misfolded regions, yellow bars demonstrate the error region between 95% and 99%, and green bars indicate the region with a lower error rate for protein folding.

A)

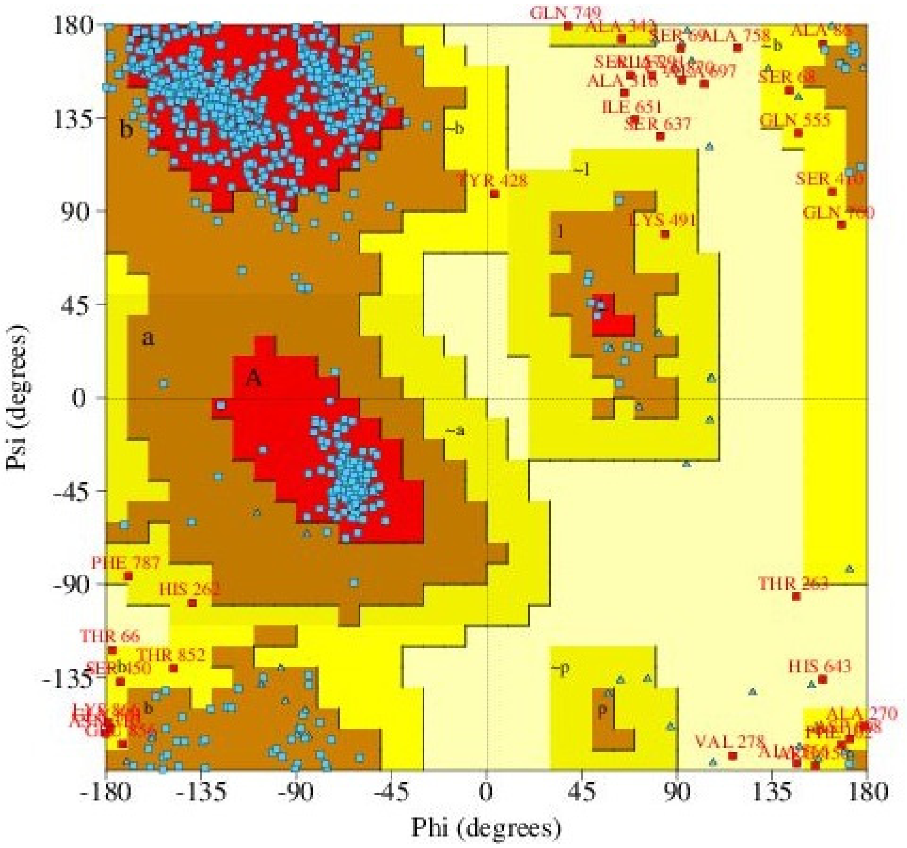

Psi (degrees)
Phi (degrees)
180
135
90
45
0
-45
-90
-135
-180
-135
-90
-45
0
45
90
135
180
GLN 749
ALA 342
ALA 758
SER 68
GLN 555
ILE 651
SER 637
TYR 428
SER 410
LYS 491
THR 263
HIS 643
ALA 270
VAL 278
PHE 787
HIS 262
THR 66
THR 852
A
a
b
~b
~a
~l
l
~p
p

B)

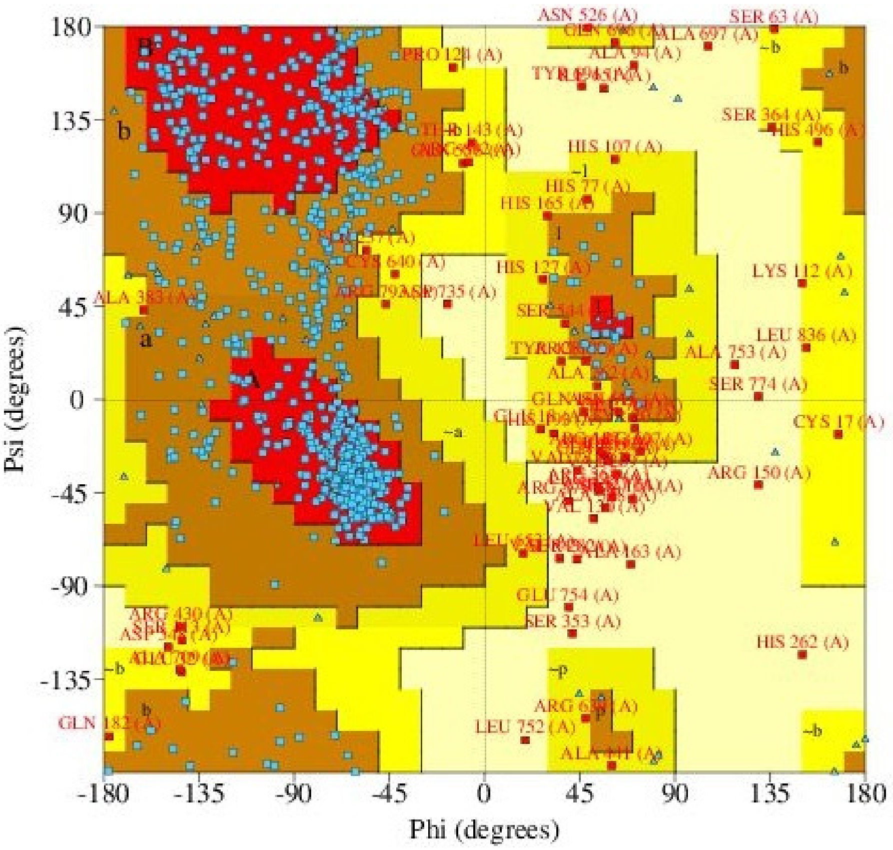

Figure S2: Ramachandran plots of the protein structures obtained via PROCHECK. A) Ramachandran plot of protein model MOD5 and B) Ramachandran plot of protein model ITAS5. The percentages of residues of model MOD5 in the most favoured regions, additionally allowed regions, generously allowed regions and disallowed regions are 79.7, 15.5, 3.0 and 1.8 %, respectively. For model ITAS5, 61.0, 29.8, 5.9 and 3.3 % of the amino acid residues were predicted to be in the most favoured, additionally allowed, generously allowed and disallowed regions, respectively.

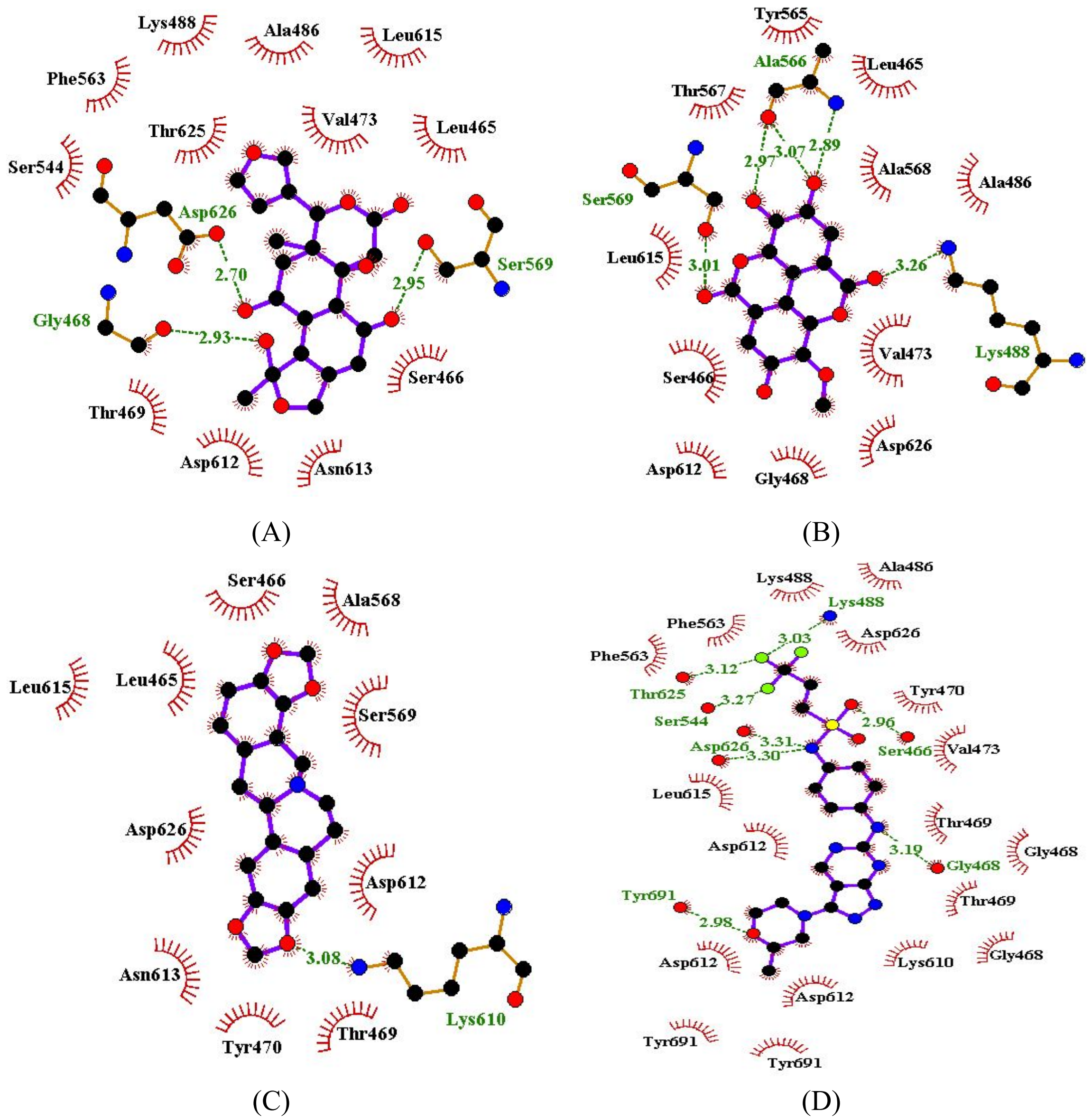

Figure S3: The 2D diagrams of the *Ld*CRK12-ligand interaction generated using LigPlot+. Interaction profiles of (A) *Ld*CRK12-ZINC000095485940 complex; (B) *Ld*CRK12-NANPDB1406 complex; (C) *Ld*CRK12-NANPDB2581 complex and (D) *Ld*CRK12-GSK3186899 complex. The ligands are coloured in purple, hydrogen bonds are represented as green dash lines and hydrophobic contacts are represented as red spoke arcs.

A)

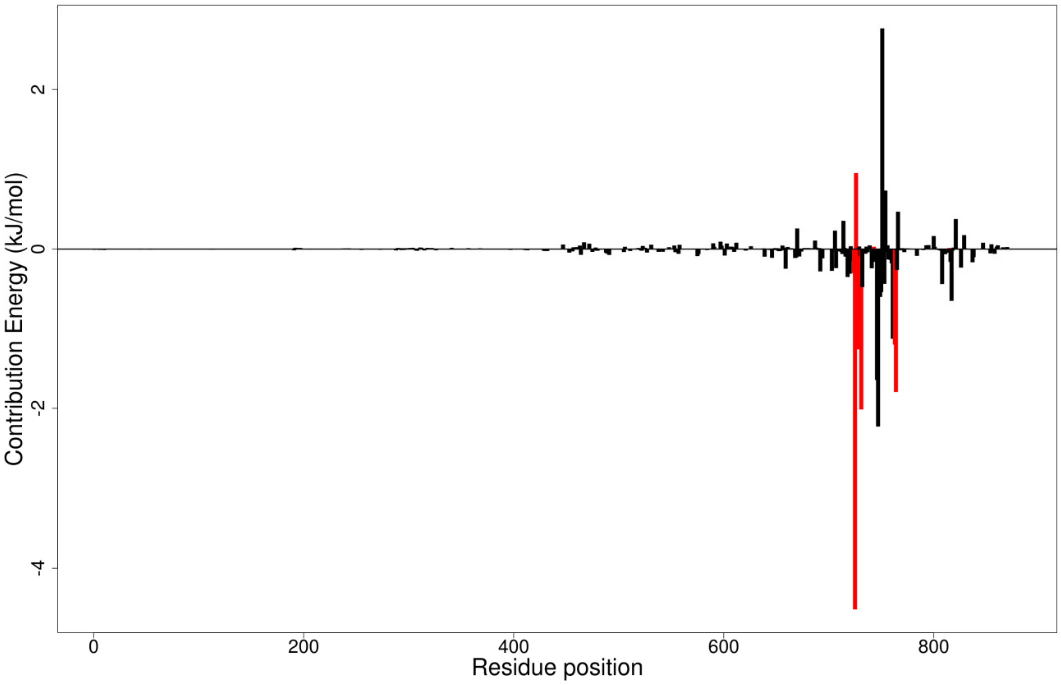

Contribution Energy (kJ/mol)
2
0
-2
-4
0
200
400
600
800
Residue position

B)

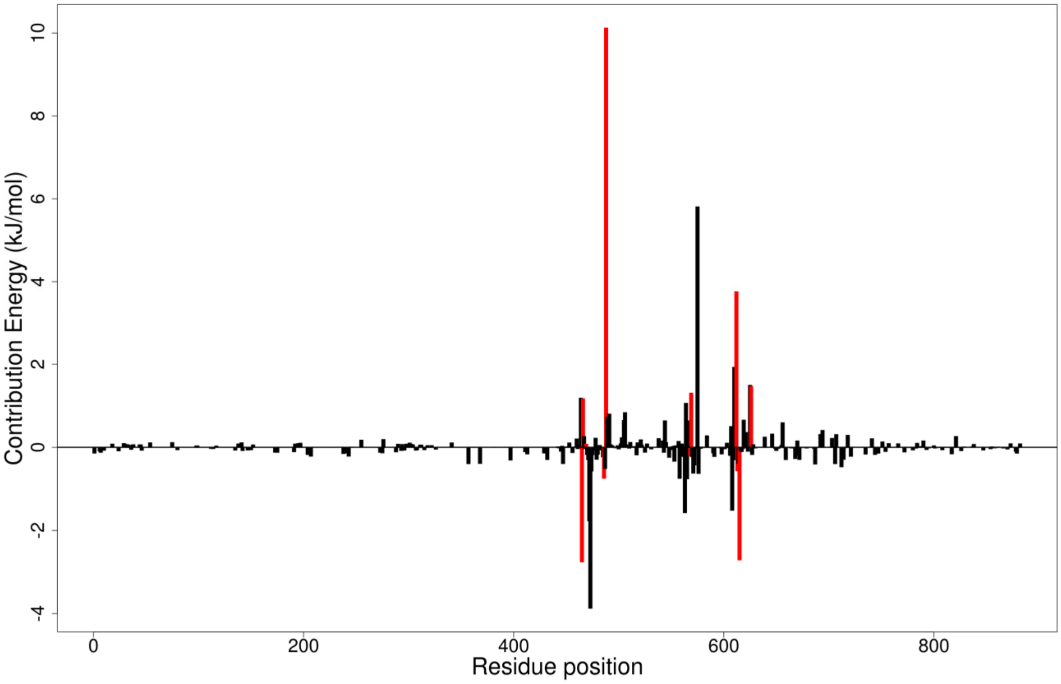

Contribution Energy (kJ/mol)
10
8
6
4
2
0
-2
-4
0
200
400
600
800
Residue position

C)

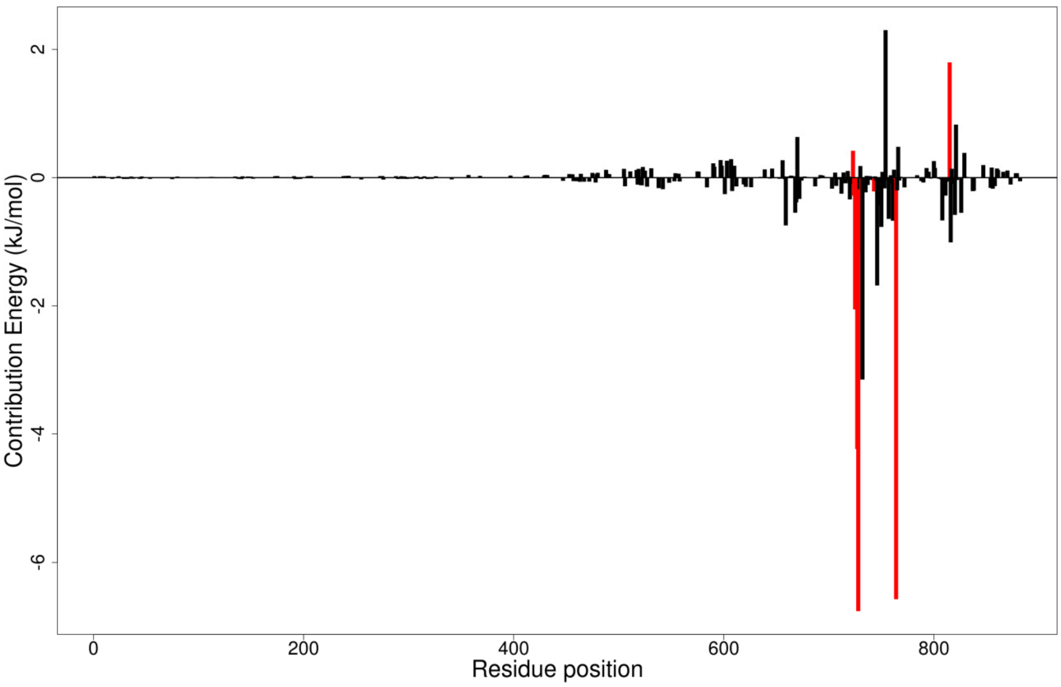
Contribution Energy (kJ/mol)
Residue position
2
0
-2
-4
-6
0
200
400
600
800

D)

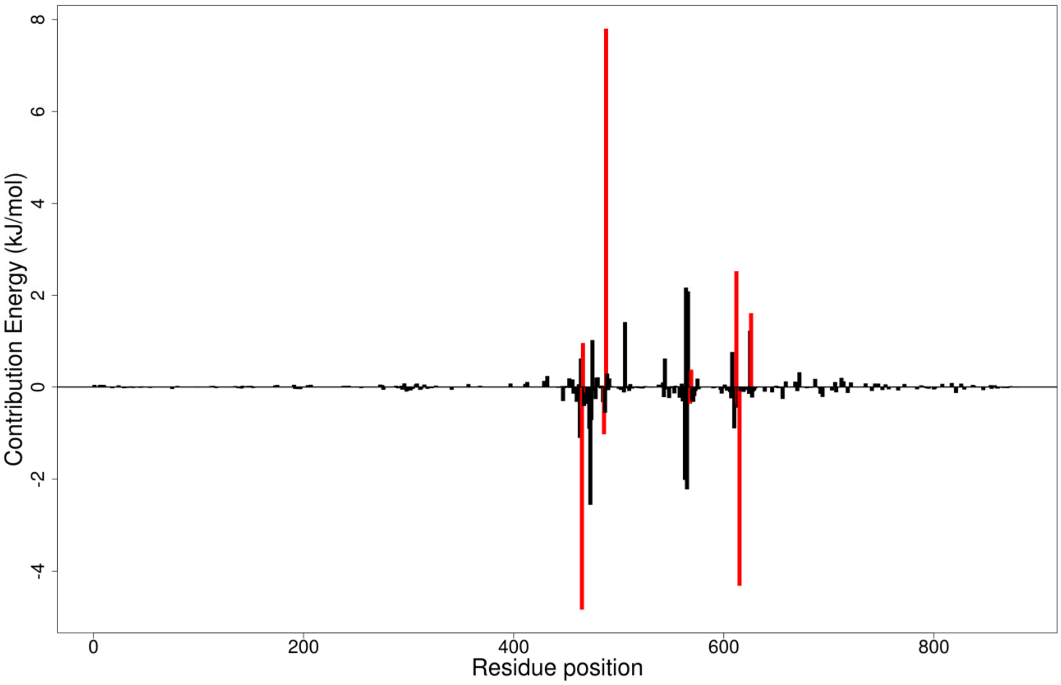
Contribution Energy (kJ/mol)
Residue position
8
6
4
2
0
-2
-4
0
200
400
600
800

E)

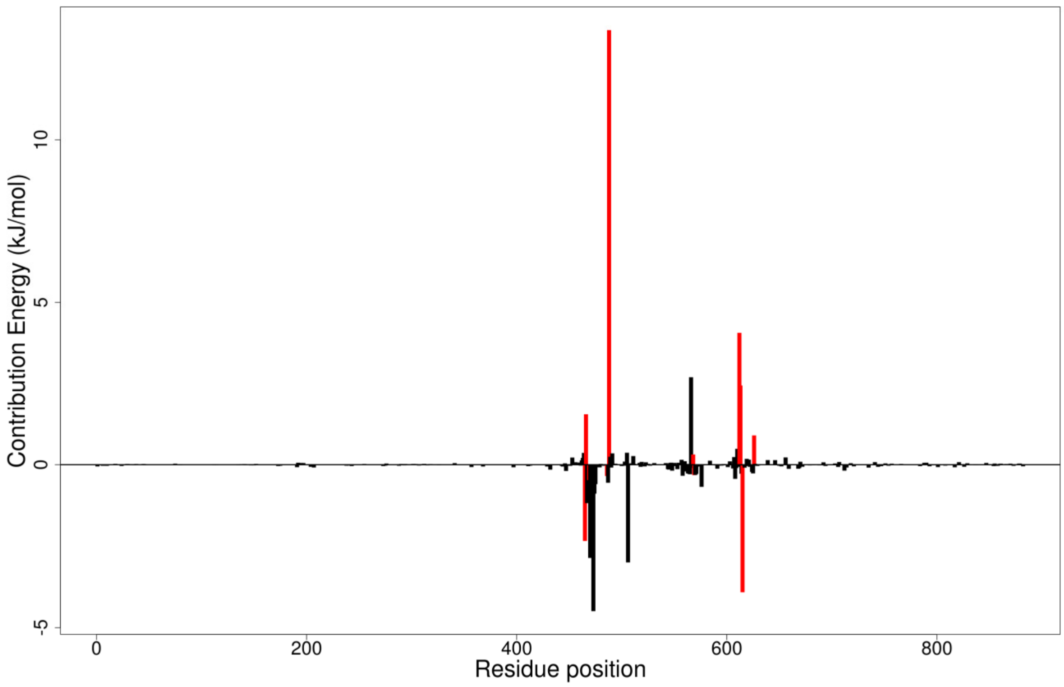

Contribution Energy (kJ/mol)
10
5
0
-5
0
200
400
600
800
Residue position

F)

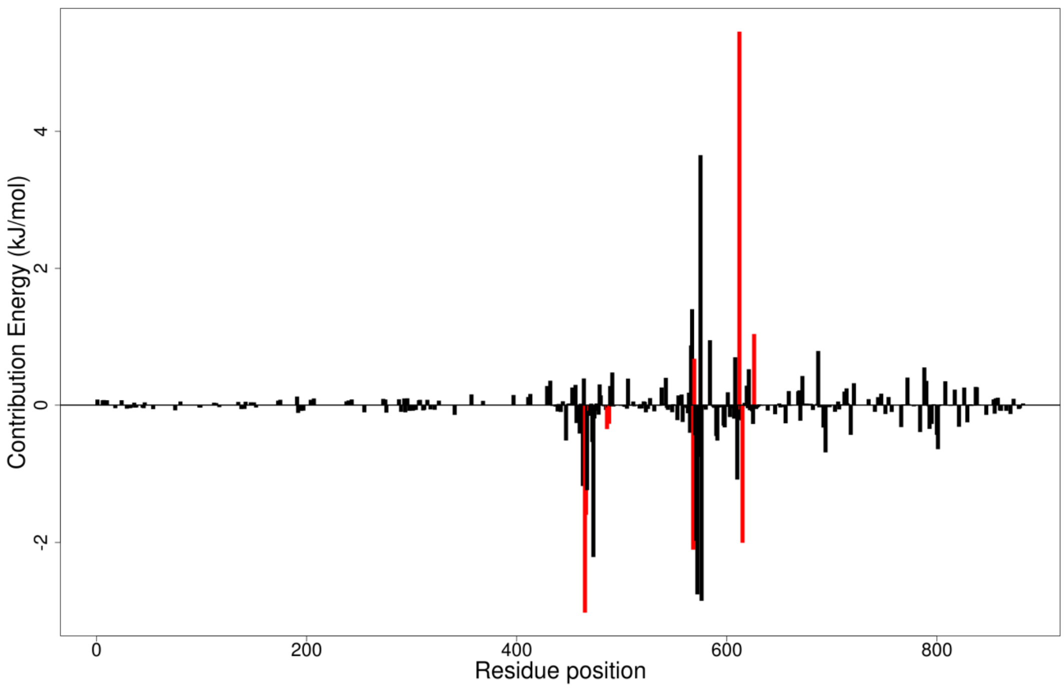

Contribution Energy (kJ/mol)
4
2
0
-2
0
200
400
600
800
Residue position

G)

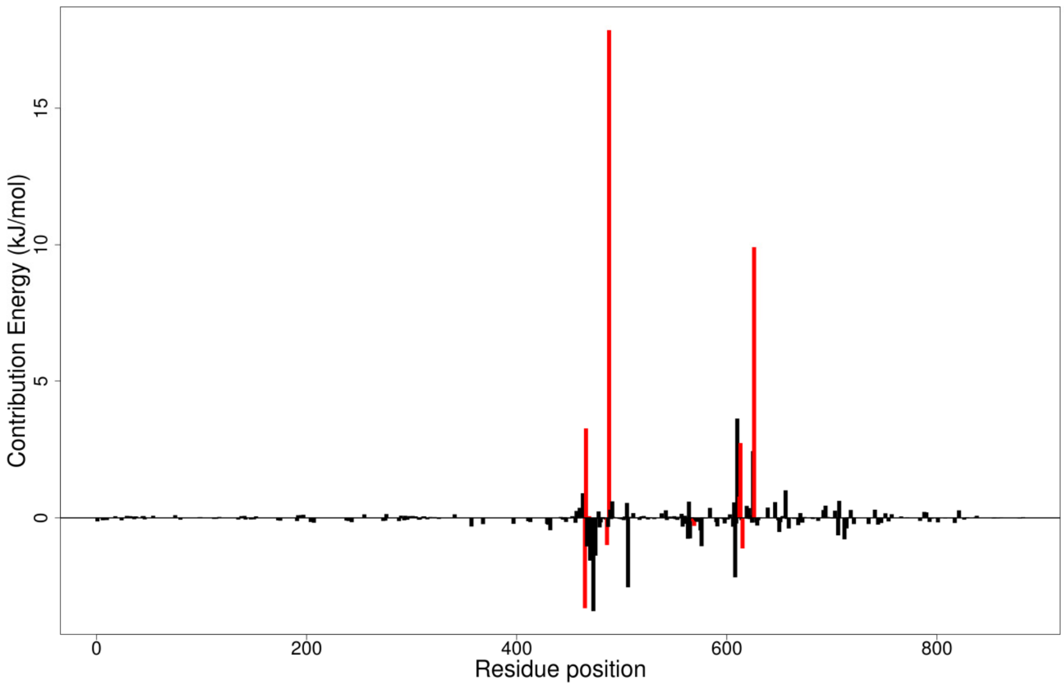

Figure S4: Molecular mechanics Poisson-Boltzmann surface area (MM-PBSA) plot of binding free energy contribution per residue of protein-ligand complexes A) *Ld*CRK12-compound 5 B) *Ld*CRK12-compound 7; C) *Ld*CRK12-compound 8; D) *Ld*CRK12-NANPDB1649 E) *Ld*CRK12-NANPDB2581 F) *Ld*CRK12-NANPDB6446 G) *Ld*CRK12-ZINC000095485940.
